# The shared neural substrates of emotional mimicry and emotional contagion: an activation likelihood estimation meta-analysis and meta-analytic connectivity modeling analysis

**DOI:** 10.1093/scan/nsaf091

**Published:** 2025-09-10

**Authors:** Yujia Fu, Dan Wang, Junye Liu, Hui Wang, Fuqi Wen, Wenfeng Chen, Zhengkui Liu

**Affiliations:** Department of Psychology, Renmin University of China, Beijing 100872, P.R. China; Department of Psychology, Renmin University of China, Beijing 100872, P.R. China; Department of Psychology, Renmin University of China, Beijing 100872, P.R. China; Department of Psychology, Renmin University of China, Beijing 100872, P.R. China; Department of Psychology, Renmin University of China, Beijing 100872, P.R. China; Faculty of Health and Wellness, City University of Macau, Macau 999078, P.R. China; Institute of Psychology, Chinese Academy of Sciences, Beijing 100101, P.R.China

**Keywords:** emotional contagion, emotional mimicry, facial mimicry, meta-analysis, functional connectivity analysis

## Abstract

Emotional contagion is an important aspect of social interaction. Traditional theories suggest that it relies on mimicry of facial or emotional movements. To address the question of whether there is a distinction between emotional contagion and emotional mimicry, we conducted a meta-analysis using the Activation Likelihood Estimation algorithm to identify brain regions activated by the two tasks. We then evaluated the co-activation patterns of these common regions using meta-analytic connectivity modelling (MACM). The results show partial overlap in brain regions, such as the cingulate gyrus, middle frontal gyrus, and inferior parietal lobule, between emotional contagion and emotional mimicry. Contrast analyses further identified distinct brain regions activated by each task. MACM analysis indicated that regions including the thalamus, putamen, precentral gyrus, and insula play critical roles in the co-activation network.

Highlights

- Emotional contagion activates a system of mirror neurones, including the inferior frontal gyrus, the inferior parietal gyrus, and the precentral gyrus.
- Although emotional contagion shows more activation than facial mimicry in the middle frontal gyrus (MFG), superior frontal gyrus (SFG), middle temporal gyrus (MTG), and parahippocampal gyrus, both tasks overlap in regions like the inferior frontal gyrus, precentral gyrus, and insula.
- Regions such as the thalamus, precentral gyrus, and insula play key roles as critical nodes in the co-activation network.

## Introduction

Imagine you are watching a movie with a friend, and you notice that your friend begins to wipe away tears. You might unconsciously feel emotionally affected and start to feel sad as well. This phenomenon is referred to as emotional contagion, which occurs when we unknowingly ‘catch’ the emotions of others, resulting in a response and mental state that align with theirs (Hatfield et al. 1993, Palagi et al. 2020). Through emotional contagion, humans are able to synchronize with the emotional states of others, which fosters unconscious resonance. This process helps promote prosocial behaviours and strengthens emotional connections (Hatfield et al. 1993, De Waal 2012, Siposova and Carpenter 2019). However, the internal emotional state of another person is not directly observable. Therefore, how we convey others’ internal emotional states through external cues, such as behaviour and facial expressions, during face-to-face interactions remains a complex and controversial issue (Goldman and de Vignemont 2009, Aviezer et al. 2017, Barrett et al. 2019).

In studies of emotional contagion, proponents of the simulation feedback mechanism argue that facial mimicry may serve as a means of achieving emotional synchronization (Hatfield et al. 1993, Preston and de Waal 2002, Prochazkova and Kret 2017). Facial mimicry refers to the process by which individuals replicate others’ facial expressions; as a form of muscular motor response, this process may be driven by the mirror neurone system (MNS) (Fischer and Hess 2017). Although facial muscle movements are not always directly associated with emotional expression or experience (Palagi et al. 2020, Hess 2021), both emotional contagion and facial mimicry may involve emotional synchronization, though potentially via distinct neural pathways.

Research has shown that during social interactions, individuals tend to automatically mimic others’ facial expressions, vocal tones, and postures, which in turn triggers similar emotional experiences in themselves. Facial mimicry partially mediates the relationship between observed emotional expressions and self-reported emotions (Olszanowski et al. 2020).

The neural mechanisms of emotional contagion are still not fully understood. Some researchers propose that the MNS may play a role in emotional contagion by triggering physiological or neural responses through synchronization with the emotional states of others (Nummenmaa et al. 2008, Franklin 2019, Paz et al. 2022). The MNS involves areas such as the inferior frontal gyrus (IFG), somatosensory cortex, premotor area, inferior parietal lobule (IPL), and superior temporal gyrus (STG) (Carr et al. 2003, Gallese et al. 2004, Braadbaart et al. 2014, Keysers and Gazzola 2014, Budell et al. 2015, Mazza et al. 2015, Rizzolatti and Caruana 2017). When individuals perceive emotional signals from others, like gestures or facial expressions, the brain automatically activates neural representations similar to those of the observed individual. These shared neural representations enable us to directly interpret the emotions of others (Gallese et al. 2004). This system is typically engaged during motor observation and is associated with reflecting others’ bodily and motor actions. But current research has not definitively mapped emotional processing to specific neural regions (Hickok 2009); these neurones might only perform basic motor associations or sensory-motor connections (Iacoboni 2007, Downey 2008).

Building on findings such as those of Nummenmaa et al. (2008), who reported increased limbic activity when participants viewed emotional faces, researchers have proposed that emotional mirroring may involve a system at least partially distinct from the classical motor MNS. In addition to regions like the IFG and premotor cortex typically associated with motor mirroring, structures such as the insula, anterior cingulate cortex (ACC), and amygdala appear to support emotional perception, interoceptive integration, and expressive output (Gallese et al. 2004, Keysers and Gazzola 2014, Rizzolatti and Caruana 2017). This limbic-centred ‘emotional mirroring system’ may allow individuals to resonate with others’ emotional states even when motor mirroring pathways are impaired (Cerniglia et al. 2019). Emotion, by nature, involves not only affect but also motor and bodily components (Bastiaansen et al. 2009). According to the embodied simulation theory, observing someone else’s emotional expressions—such as a disgusted face—can evoke similar visceral responses in the observer (Carr et al. 2003, Wicker et al. 2003, De Vignemont and Singer 2006, Schurz et al. 2021, Keysers and Gazzola 2023). Neuroimaging studies show that this process recruits both limbic regions and motor-related areas like the posterior parietal cortex (Carr et al. 2003, Liu et al. 2021, Xu et al. 2021). Among these, the insula may serve as a central hub linking interoceptive-emotional states with motor representations (Carr et al. 2003, Caruana 2019, Evrard 2019, Del Vecchio et al. 2024), suggesting a dynamic integration between emotional resonance and embodied expression.

However, the specific role of the MNS in emotional contagion remains unclear (Hickok 2009). The ventral premotor cortex (vPMC) and IFG (Hennenlotter et al. 2005, Van der Gaag et al. 2007), the supplementary motor area (SMA) (Decety et al. 2008), and the inferior parietal lobe (Plata-Bello et al. 2023) all show activation during the observation of emotions and pain perception. Disruption of the MNS can impair emotion recognition (Keysers et al. 2018). These findings suggest that the specific role of the MNS in emotional contagion remains to be clarified.

The embodied emotion model suggests that individuals can generate the same emotional experience by simulating their own facial expressions in similar situations (Gallese et al. 2004, Gallese 2009, Danish et al. 2020, Farina 2021). Therefore, emotional contagion and facial mimicry are neurologically linked (Hirsch et al. 2023). However, at the behavioural level, emotional contagion and mimicry appear to be dissociated (Hess and Blairy 2001, Van der Schalk et al. 2011). Hess and Blairy (2001) conducted a study in which participants watched a video of dynamic facial expressions without explicit instructions to mimic them. The study found no direct correlation between the participants’ facial muscle mimicry and their emotional responses to the video. Although mimicry is typically associated with emotional contagion, the causal relationship between facial expressions and subjective emotional experience remains complex (Olszanowski et al. 2020). At the group level, compared to emotional contagion, mimicry is influenced by other social contextual factors, as evidenced by the effect of facial self-similarity on mimicry (Olszanowski et al. 2022). Van der Schalk et al. (2011) tested the correlation between facial behaviours and self-reported feelings in response to photos or videos but found no significant link between mimicry and contagion. This finding suggests that mimicry may have a social component, functioning as a mechanism to enhance affinity and bonding within a group, rather than serving as a direct driver of emotional contagion in all contexts.

In summary, the neural systems underlying facial mimicry and emotional contagion appear to be distinct, and changes in facial expressions do not necessarily lead to changes in subjective emotional experience. The two may occur simultaneously in real-life interactions. The complexity of emotional contagion and emotional mimicry may arise from inconsistent experimental results, differences in researchers’ definitions, and limitations in experimental materials. Therefore, conducting a brain imaging meta-analysis to distinguish the neural mechanisms of the two is necessary. The aim of the study is to differentiate emotional contagion from emotional mimicry. It is hypothesized that the MNS may mediate emotional contagion through a simulation mechanism, with varying levels of involvement in emotional contagion and emotional mimicry.

To achieve this objective, we first employ the Activation Likelihood Estimation (ALE) method to conduct two quantitative meta-analyses of existing functional magnetic resonance imaging (fMRI) studies, distinguishing brain regions activated by emotional contagion and mimicry. Then, by comparing and jointly analysing the results of both, we use meta-analytic connectivity modelling (MACM) to identify co-activation patterns in shared brain regions. Given the scarcity of neuroimaging studies on emotional contagion, sample heterogeneity and the diversity of experimental paradigms may influence the meta-analysis results (Müller et al. 2018). To balance sample heterogeneity with robustness, we aim to include as many relevant studies as possible while maintaining consistent research standards to avoid biased results and ensure the effectiveness and robustness of the ALE analysis (Eickhoff et al. 2016).

## Materials and methods

### Literature search and study selection

To select the literature related to emotional mimicry, we searched the literature through the following databases before 6 September 2023: Web of Science, PubMed (https://pubmed.ncbi.nlm.nih.gov/), Google Scholar, PsycINFO, and JSTOR.

The keywords involved in *emotional mimicry* were ‘emotional imitation’ AND ‘fMRI’ OR ‘functional magnetic resonance imaging’, OR ‘emotional mimicry’ AND ‘fMRI’ OR ‘functional magnetic resonance imaging’, OR ‘facial imitation’ AND ‘fMRI’ OR ‘functional magnetic resonance imaging’, OR ‘facial mimicry’ AND ‘fMRI’ OR ‘functional magnetic resonance imaging’.

For the articles on *emotional contagion*, we used keywords as follows: ‘emotional observation’ AND ‘fMRI’ OR ‘functional magnetic resonance imaging’, OR ‘emotional contagion’ AND ‘fMRI’ OR ‘functional magnetic resonance imaging’, OR ‘affective sharing’ AND ‘fMRI’ OR ‘functional magnetic resonance imaging’, OR ‘emotional empathy’ AND ‘fMRI’ OR ‘functional magnetic resonance imaging’, OR ‘affective empathy’ AND ‘fMRI’ OR ‘functional magnetic resonance imaging’.

The criteria that we employed to select the appropriate research for this review included: (i) All participants were mentally and physically healthy, or the research contained the control condition using normal participants and separately reported the effect of healthy participants in the baseline group; (ii) measurement of blood oxygenation through fMRI; (iii) the fMRI studies using whole-brain group analysis, not region-of-interest (ROI), which was to ensure that the likelihood of activation under the null hypothesis is equal across the brain (Eickhoff et al. 2009); (iv) the standard stereotactic space used to spatially align imaging data was Montreal Neurological Institute (MNI) or Talairach; (v) the experimental stimuli were static pictures of the whole face or dynamic video clips of the whole face or real-time interactive imaging; (vi) the study should involve basic emotions, with the experimental tasks focusing on mimicking facial expressions rather than meaningless facial movements or mimicking emotional meanings. During observation, there should be no emphasis on mimicking a specific target; (vii) the baseline groups in the emotional mimicry or contagion task involve the following conditions: (a) Mimicking or observing with the neutral face; (b) making a simple judgement about the attribute of the stimuli; and (viii) activations, rather than deactivations, were included. Additionally, as the studies included span from 2003 to 2023, self-reports may be influenced by measurement implementation, social desirability effects, and cultural differences. Therefore, we did not use the presence or absence of self-report measures as a criterion for inclusion or exclusion of studies.

**Figure 1.**
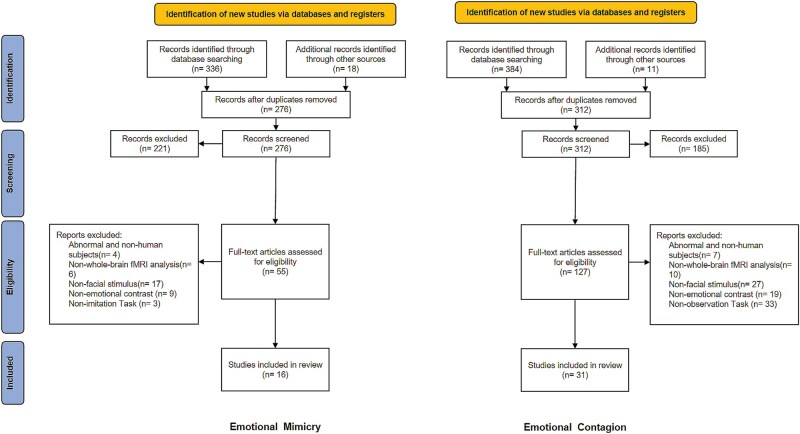
Procedure of the study selection.

According to the above criteria, each author independently screens the titles and abstracts of the retrieved articles to remove irrelevant studies and inappropriate article types (e.g. reviews). In this step, each author screens all entries and cross-validates to ensure that all relevant articles are identified according to the Preferred Reporting Items for Systematic Review and Meta-Analysis Protocols (PRISMA-P) 2020 statement. Then, the eligibility of all articles in the previous step was assessed by full-text screening, with two authors independently assessing all articles, removing those that did not fit and explaining why, and then comparing the results of their screening to make a final decision on which articles to keep. The specific screening processes of the article are shown in Fig. 1. Detailed information, such as the author, number of participants, and their mean age, and extracted coordinate values of MNI or Talairach were also recorded (Table S1, see online supplementary material for a colour version of this table).

### Data extraction

All data were extracted by two authors from all selected studies and cross-validated by one additional author. We extracted focal coordinates, sample size, and age information from the conforming studies. The discrete emotions that emerged from interest comparisons in the conforming studies were mostly basic emotions (happiness, fear, sadness, anger, disgust), and individuals with complex emotions included in some of the studies were not included in the analysis. To avoid sample overlap, only one contrast was selected for each included study, and for studies that contrasted focused emotions, we selected as many emotion groupings and under-represented emotion contrasts as possible, e.g. fewer fear > neutral contrasts emerged relative to anger > neutral, so the preferred emotion grouping of fear > neutral extracted coordinates. All extracted coordinates were converted to MNI by full use of the algorithm implemented in GingerALE for the studies reported in Talairach space (Lancaster et al. 2007, Laird et al. 2009).

### Meta-analyses

In this study, our focus was to identify the brain regions and the variations engaged in two distinct mental processes. We employed a multifaceted approach, incorporating single analysis, contrast analysis, overlap analysis, and meta-analytic connectivity mapping (MACM) analysis. The single analysis of ALE discovered the brain regions associated with two domains individually. Through contrast analysis of ALE, we were able to distinguish the brain regions unique to two domains, highlighting domain-specific systems and identifying regions common to two behavioural domains. Conjunction analysis facilitated the discovery of regions shared across two domains. Additionally, MACM analysis was utilized to investigate the potential networks common to the processes, focusing on regions identified as overlaps across the two domains.

#### Activation Likelihood Estimation

ALE can be used to identify reliable activation patterns in 3D space across studies by calculating the probability of at least one activated focus located in voxels and determining the ALE value. The ALE score is subsequently juxtaposed with the probability of activation for a standard space voxel, computed using a null distribution derived analytically for random spatial correlation across experiments. Thus, the ALE algorithm facilitates the assessment of whether the merging of experiments from various studies indicates significant processes instead of arbitrary grouping in the brain (Turkeltaub et al. 2002, Eickhoff et al. 2009).

We extracted the experimental information and coordinated data from the studies that met the selective criteria and generated a text file. We performed a series of ALE meta-analyses in MNI’s standard space using Ginger ALE v3.0.2 (https://www.brainmap.org/ale/). All coordinates reported in Talairach format in 47 studies were converted into MNI space. Based on the selection criteria, emotional mimicry was included in 16 peer-reviewed neuroimaging articles and emotional contagion in 31 articles for meta-analysis (see Table S1 for lists of included studies, see online supplementary material for a colour version of this table). These studies were published between 2003 and 2023. Detailed information, such as the author, number of participants, and their mean age, and extracted coordinate values of MNI or Talairach were also recorded.

We use cluster-level family-wise error (FWE) to correct *P*-value images, where the FWE threshold is set to .05, threshold permutations are set to 5000, and the cluster-forming threshold is set to *P* < .001.

#### Conjunction and contrast analyses of emotional mimicry and emotional contagion

Contrast analysis was conducted to compare emotional mimicry and contagion using GingerALE v3.0.2. Data files from two selected studies were merged into a single file for a combined analysis. The analysis utilized a cluster inclusion criterion set at a voxel-level significance of *P* < .001, applying FWE correction at the cluster level to achieve a significance threshold of *P* < .05. A conjunction image was created highlighting shared brain regions identified in both studies (Eickhoff et al. 2011). Differences between the two ALE maps were assessed using permutation tests to evaluate the significance of ALE score differences, with a significance threshold set at *P* < .01, 5000 permutations. The resulting *P*-value map was then converted to *z*-scores for visualization.

To identify shared brain regions between the two domains, we utilized Mango v4.1 (https://mangoviewer.com/) to visualize the overlap of thresholded statistical parametric maps from two single analyses on emotional mimicry and emotional contagion.

#### Meta-analytic connectivity modelling

This approach posits that voxels demonstrating co-activation beyond random chance in fMRI studies are functionally interconnected. Initially, the intersection of brain regions identified from the overlap analysis was designated as volumes of interest (VOI). We pinpointed four primary VOIs from the conjunction of significant cluster-level findings in tasks related to emotional mimicry and contagion (Table 1). The MACM leveraged a neuroimaging database to outline co-activation patterns across various tasks, creating co-activation maps from predetermined VOIs (Langner et al. 2014), which facilitated the mapping of the emotional contagion circuit and evaluation of each important network within this neural loop.

**Table 1.** Summary of the location-based search in the BrainMap database of the four VOIs for MACM analyses.

VOI	Number of experiments	Number of participants	Number of foci
Cluster 1 (left cingulate)	109	1266	6163
Cluster 2 (right MFG)	57	1002	3642
Cluster 3 (right IPL)	29	368	1460
Cluster 4 (right PreG)	20	331	1152

For sourcing neuroimaging studies that highlighted activations in these seed regions, we utilized the BrainMap database through Sleuth v3.0.4 (https://www.brainmap.org/tools.html), downloading activated coordinates in MNI format. Our search was restricted to ‘Normal Mapping’ with an ‘Activation Only’ condition in healthy subjects, excluding studies focusing on differences due to age, gender, interventions, or clinical conditions. Subsequently, an ALE meta-analysis was conducted on the coordinates from these experiments to measure their convergence and co-activation with the ROIs. The resulting ALE map underwent correction for FWE at *P* < .05 at the cluster level.

#### Publication bias accessing

Considering that brain imaging studies also suffer from file drawer problems, there are still some studies that fail to be published because they do not obtain the expected results, leading to overestimation of meta-analysis effect sizes. We refer to the methodology of Acar et al. (2018) to measure whether the articles we included in the meta-analysis were at risk of publication bias in the mimicry and observation tasks, respectively. The rationale for this approach is to add studies that do not report a focus of activation in the target region to the existing meta-analysis to determine the amount of counter-evidence FailSafe N (FSN) required before the convergence of the focus space of studies in that region is no longer statistically significant, with larger FSN values indicating more stable and robust clustering results. All the codes used in the analysis process can be found on GitHub (https://github.com/NeuroStat/FailSafeN). First, the noise studies were generated with the R code, saving the spatial coordinates of the sample sizes and the number of peaks and creating new text files by pasting the required number of noise studies after the original meta-analysis studies, feeding them into GingerALE 2.3.6 to perform the meta-analysis, and repeating the iterations until the FSNs of all clusters were calculated. The FWE threshold is set to 0.05, threshold permutations are set to 1000, and the cluster-forming threshold is set to *P* < .01.

## Results

### Publication bias

Predefining the FSN minimizes the number of meta-analysis runs. For brain imaging studies, the 95% confidence interval for the number of studies reporting no local maxima is 5–30 studies per 100 studies, so we predefined the minimum FSN values as 5 and 9 studies for the emotional mimicry and emotional contagion tasks, respectively. We aimed that for each cluster, there should be at least 10% of studies contributing, thus determining the maximum FSN value for each cluster. Since the general range of FSN is defined as 2*k*–10*k*, where *k* is the number of studies included in the meta-analysis, we also considered the threshold setting of the internal FSN. We show the robustness of each cluster activation in Table 2.

**Table 2.** Properties and FSN of the clusters resulting from the meta-analysis.

Cluster number	Volume (mm^3^)	Weighted centre (*x*, *y*, *z*)			Number of contributing studies	Predefined boundaries of FSN	FSN
Emotional mimicry							
1	2904	−50	8	6	8	[5, 72]	≥72
2	2832	−52	−6	38	10	[5, 90]	≥90
3	2648	4	−4	62	9	[5, 81]	24
4	2512	46	−10	36	10	[5, 90]	≥90
5	1912	−2	10	42	8	[5, 72]	24
6	1680	56	6	14	7	[5, 63]	61
7	800	56	−28	24	5	[5, 45]	24
Emotional contagion							
1	3472	−44	24	−12	14	[9, 126]	20
2	2576	54	30	−4	13	[9, 117]	58
3	2192	50	−36	4	10	[9, 90]	≤9
4	2024	20	−4	−18	12	[9, 108]	17
5	1840	−40	−76	−14	9	[9, 81]	35
6	1608	−10	54	30	8	[9, 72]	16
7	1448	−50	−58	8	7	[9, 63]	61
8	1304	8	12	64	8	[9, 72]	60
9	1280	−20	−6	−16	7	[9, 63]	≤9
10	1272	36	−56	−12	9	[9, 81]	≥81
11	1264	−4	18	48	8	[9, 72]	61
12	1040	44	18	−36	6	[9, 54]	57
13	792	10	−30	−2	8	[9, 72]	17
14	744	58	−30	26	5	[9, 45]	≤9
15	704	52	2	46	4	[9, 36]	≤9

### ALE results

**Figure 2.**
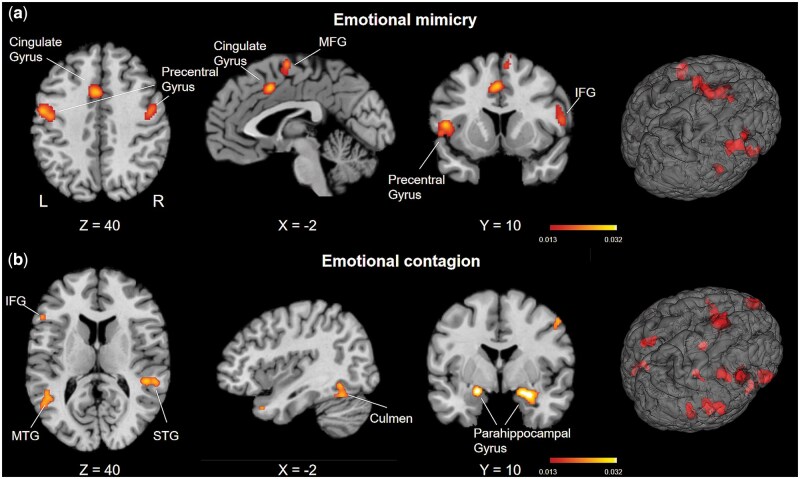
Regions activated by emotional contagion and emotional mimicry. (a) Regions activated by emotional mimicry. These regions include pars opercularis of the right IFG (BA44), middle frontal gyrus (MFG), bilateral precentral gyrus (PreG), left STG, and left insula, projected onto a standard MNI surface at an *P* < .05 threshold. (b) Regions activated by emotional contagion. These regions include bilateral IFG (pars orbitalis of left IFG, BA47; pars triangularis of right IFG, BA45), right precentral gyrus (PreG), bilateral superior temporal gyrus (STG), left insula, and bilateral superior frontal gyrus (SFG), projected onto the same template and threshold for visualization. Clustering threshold: *P* < .05 (FWE correction for clustering level). Coordinates are MNI152 standard stereotaxic space. Figures were created using Mango (http://ric.uthscsa.edu/mango).

For the emotional mimicry task, 16 experimental contrasts across 314 subjects were included in the meta-analysis. The results demonstrated that emotional mimicry induced greater activation in the pars opercularis of the right IFG (BA44), middle frontal gyrus (MFG), bilateral precentral gyrus (PreG), left STG, left insula, bilateral medial frontal gyrus, right IPL, and bilateral cingulate gyrus (Fig. 2, Table 3).

**Table 3.** Activation convergence results of meta-analysis.

Cluster	Volume (mm^3^)	Hemisphere	Region	BA(s)	Coordinate			ALE (×10^−2^)
					*x*	*y*	*z*	
Emotional mimicry								
1	2904	Left	Precentral gyrus	44	−50	8	6	3.07
		Left	Superior temporal gyrus	22	−54	12	−2	2.18
		Left	Insula	13	−40	−2	6	1.57
2	2832	Left	Precentral gyrus	6	−52	−6	38	3.10
3	2648	Right	Medial frontal gyrus	6	4	−4	62	2.98
		Left	Medial frontal gyrus	6	−2	−6	64	2.33
		Right	Medial frontal gyrus	6	6	8	60	2.03
4	2512	Right	Precentral gyrus	6	46	−10	36	3.19
		Right	Middle frontal gyrus	6	46	2	48	2.04
5	1912	Left	Cingulate gyrus	24	−2	10	42	2.80
		Right	Cingulate gyrus	32	6	18	34	1.75
6	1680	Right	Inferior frontal gyrus	44	56	6	14	2.23
7	800	Right	Inferior parietal lobule	40	56	−28	24	2.29
Emotional contagion								
1	3472	Left	Inferior frontal gyrus	47	−44	24	−12	3.67
		Left	Inferior frontal gyrus	47	−40	30	−16	3.32
		Left	Insula		−38	22	−6	3.01
		Left	Inferior frontal gyrus	45	−50	28	−2	2.48
		Left	Inferior frontal gyrus	45	−52	24	12	2.43
2	2576	Right	Inferior frontal gyrus	45	54	30	−4	4.88
		Right	Precentral gyrus	44	56	16	0	2.05
3	2192	Right	Superior temporal gyrus	41	50	−36	4	4.62
4	2024	Right	Parahippocampal gyrus	28	20	−4	−18	4.03
		Right	Parahippocampal gyrus		26	−2	−20	3.71
5	1840	Left	Declive		−40	−76	−14	2.95
		Left	Inferior occipital gyrus	18	−34	−88	−4	2.89
		Left	Inferior occipital gyrus	18	−30	−90	−2	2.88
		Left	Declive		−36	−76	−18	2.73
6	1608	Left	Superior frontal gyrus	8	−10	54	30	3.32
		Left	Superior frontal gyrus	8	−8	54	40	2.35
7	1448	Left	Middle temporal gyrus	37	−50	−58	8	3.07
		Left	Superior temporal gyrus	39	−48	−52	8	2.83
8	1304	Right	Superior frontal gyrus	6	8	12	64	3.67
		Right	Superior frontal gyrus	6	4	12	54	2.22
9	1280	Left	Parahippocampal gyrus		−20	−6	−16	4.72
10	1272	Right	Declive		36	−56	−12	3.08
		Right	Culmen		40	−58	−22	2.61
11	1264	Left	Superior frontal gyrus	6	−4	18	48	2.76
		Left	Cingulate gyrus	32	−6	12	42	2.64
12	1040	Right	Superior temporal gyrus	38	44	18	−36	3.43
		Right	Superior temporal gyrus	38	48	10	−36	2.90
13	792	Right	Thalamus		10	−30	−2	3.87
		Right	Thalamus		22	−28	−4	2.29
14	744	Right	Inferior parietal lobule	40	58	−30	26	2.76
		Right	Postcentral gyrus	40	66	−28	22	2.74
		Right	Inferior parietal lobule	40	64	−22	24	2.17
15	704	Right	Precentral gyrus	6	52	2	46	3.73

For the emotional contagion, 31 experimental contrasts across 1095 subjects were included in the meta-analysis. The results demonstrated that emotional observation induced greater activation in the bilateral IFG pars orbitalis of the left IFG (BA47) and pars triangularis of the right IFG (BA45), right PreG, bilateral STG, left insula, bilateral superior frontal gyrus (SFG), left middle temporal gyrus (MTG), right IPL, right postcentral gyrus, right culmen, bilateral declive, right thalamus, left cingulate gyrus, bilateral parahippocampal gyrus, and left inferior occipital gyrus (Fig. 2, Table 3).

### Conjunction and contrast analyses

**Figure 3.**
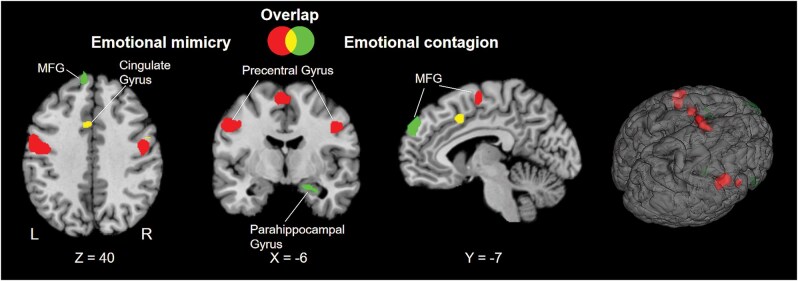
Pairwise conjunction and contrast analysis results of the two domains. Clusters are thresholded at *P* < .01, with a minimum cluster size of 200 mm^3^. L, left; R, right.

The pairwise conjunction and contrast results are presented in Fig. 3. For cluster information, please see Table 4.

**Table 4.** Clusters and peak coordinates of the ALE contrast meta-analysis.

Cluster	Volume (mm^3^)	Hemisphere	Region	BA(s)	Coordinate			*Z*
					*x*	*y*	*z*	
Emotional mimicry > emotional contagion								
1	2360	Left	Precentral gyrus	6	−53	−7.5	34.6	3.72
		Left	Precentral gyrus	6	−41	−10	38	3.24
2	1360	Right	Precentral gyrus	6	48.6	−11.5	31.2	3.72
3	1296	Left	Medial frontal gyrus	6	−4	−7	60	3.72
		Right	Medial frontal gyrus	6	6	−2	62	2.16
		Right	Medial frontal gyrus	6	8	2	56	1.89
4	984	Left	Superior temporal gyrus	22	−54	8	−4	2.40
		Left	Insula	13	−44	2	8	2.26
		Left	Superior temporal gyrus	22	−52	4	−4	2.19
		Left	Insula	13	−44	6	2	1.99
5	472	Right	Precentral gyrus	6	52	6	24	2.77
Emotional contagion > emotional mimicry								
1	1416	Left	Inferior frontal gyrus	47	−38	24	−16	3.04
		Left	Inferior frontal gyrus	47	−37	30	−16	2.67
2	1384	Left	Superior frontal gyrus	9	−16	58	29	2.79
		Left	Superior frontal gyrus	8	−10.7	60	34	2.67
		Left	Medial frontal gyrus	8	−6	56	39	2.67
		Left	Medial frontal gyrus	9	−6	61	30	2.64
		Left	Superior frontal gyrus	8	−10	52	42	2.46
		Left	Superior frontal gyrus	8	−12	50	38	2.28
		Left	Superior frontal gyrus	9	−10	56	26	2.27
3	800	Right	Inferior frontal gyrus	45	60	26	−4	2.43
		Right	Inferior frontal gyrus	45	60	32	2	2.07
4	736	Right	Middle temporal gyrus		56	−34	−4	2.69
5	704	Right	Superior temporal gyrus	38	50	12	−32	2.23
		Right	Middle temporal gyrus	21	52	6	−36	2.01
		Right	Superior temporal gyrus	38	48	16	−32	1.89
		Right	Superior temporal gyrus	38	48	20	−36	1.70
6	672	Right	Parahippocampal gyrus		20	−6	−22	2.46
		Right	Parahippocampal gyrus		26	−6	−24	2.39
		Right	Parahippocampal gyrus		34	−4	−22	1.91
7	240	Left	Inferior frontal gyrus	45	−53	25	14	2.05
		Left	Inferior frontal gyrus	45	−54	26	6	1.88

MNI, Montreal Neurological Institute.

#### Comparison between emotional mimicry and emotional contagion

As seen in Fig. 3, the mimicked process involved increased activation in the bilateral PreG, left STG, left insula, and bilateral medial frontal gyrus compared to the emotional contagion. While the emotion contagion involved more activations in the bilateral IFG (BA47, BA45), left SFG, left medial frontal gyrus, right MTG, right STG, and right parahippocampal gyrus compared to the emotional mimicry (see Table 4 and Fig. 3).

#### Overlap between the two domains

The overlap analysis between the two domains revealed clusters in the left cingulate, right MFG, and right IPL as the common area underlying emotional mimicry and emotional contagion, as shown in Fig. 3.

### MACM results

**Figure 4.**
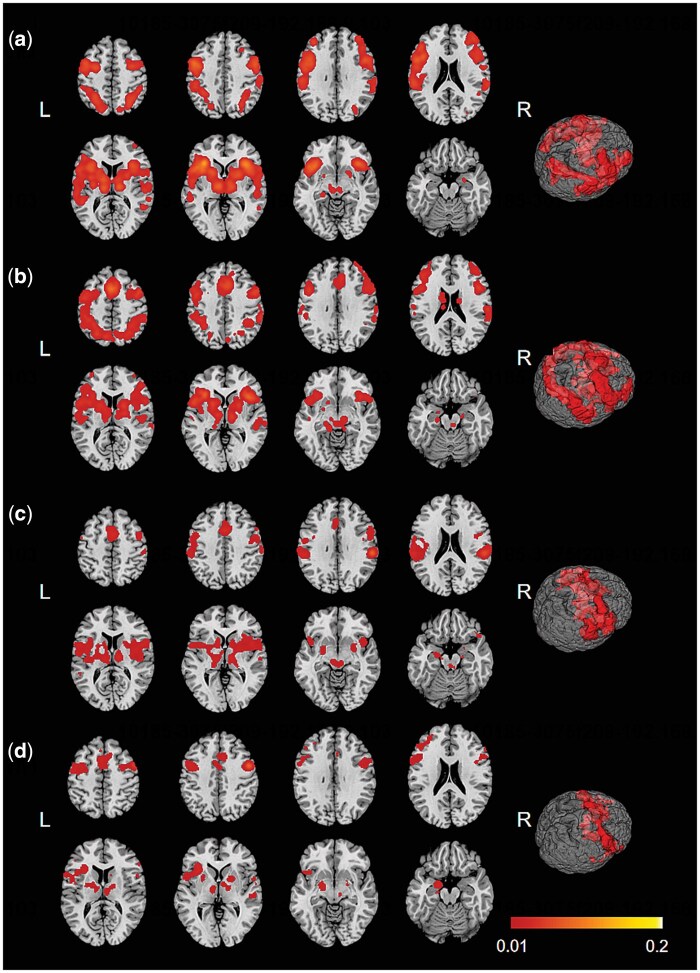
Connectivity maps of the MACM analyses. Co-activation patterns for left cingulate (a), right MFG (b), right IPL (c), and right precentral gyrus (d). Clusters threshold: *P* < .05 (cluster-level FWE correction). Coordinates are MNI152 standard stereotaxic spaces. Figure was created using Mango (http://ric.uthscsa.edu/mango). L, left; R, right.

To understand the network properties and functional connections of brain regions associated with emotional mimicry and contagion, we performed MACM analysis to reveal co-activation patterns of four VOIs (Table 1, Fig. 4), which included the left cingulate gyrus (Cluster 1), right MFG (Cluster 2), right IPL (Cluster 3), and right PreG (Cluster 4). The left cingulate showed a co-activation pattern with the bilateral insula, bilateral thalamus, bilateral PreG, right SFG, bilateral IFG (BA44), bilateral MTG, bilateral MFG, bilateral precuneus, bilateral lentiform nucleus, bilateral superior parietal lobule, right postcentral gyrus, and bilateral IPL (Table S2, see online supplementary material for a colour version of this table). For the right MFG, the co-activation patterns were found in the bilateral thalamus, left PreG, bilateral lentiform nucleus, bilateral MFG, bilateral IPL, bilateral insula, bilateral precuneus, bilateral IFG (BA9), right cingulate gyrus, and bilateral superior parietal lobule (Table S3, see online supplementary material for a colour version of this table). For the right IPL, the co-activation patterns were observed in the bilateral lentiform, bilateral thalamus, bilateral insula, bilateral PreG, bilateral IPL, bilateral STG, left cingulate gyrus, and right MFG (Table S4, see online supplementary material for a colour version of this table). The right PreG co-activated with the bilateral lentiform nucleus, bilateral thalamus, left PreG, bilateral MFG, right IFG (BA9, BA44), left insula, left parahippocampal gyrus, right STG, right cingulate gyrus, and left SFG (Table S5, see online supplementary material for a colour version of this table).

## Discussion

In this meta-analysis, we aim to elucidate the neural mechanisms underlying emotional contagion and emotional facial mimicry. Our main findings revealed that: (i) left cingulate gyrus, right MFG, and right inferior parietal gyrus are involved in emotional mimicry and emotional contagion; and (ii) bilateral thalamus, bilateral lentiform, left PreG, bilateral insula, and bilateral MFG are recruited as key nodes of the co-activation network.

### The neural basis of emotional contagion

Our single meta-analysis comparing tasks of emotional contagion revealed activation patterns across multiple brain regions, including the IFG (BA47/BA45/BA44), PreG, STG, MFG, IPL, insula, and cingulate gyrus. This suggests that emotional contagion not only relies on the motor mirror network system but also involves the limbic system.

The traditional motor MNS primarily recruits the IFG, somatosensory area, premotor area, IPL, and STG (Braadbaart et al. 2014, Binder et al. 2017, Li et al. 2020, Errante et al. 2021). Among these, the frontoparietal network (premotor area and IPL) is activated both during action execution and observation (Rizzolatti and Sinigaglia 2010, Schmidt et al. 2021, Zarka et al. 2022).

Regarding the role of the MNS in emotional contagion, studies suggest that it may primarily be responsible for the interpretation of facial muscle movement features rather than directly processing emotional valence. For example, Caruana (2022) found that vPMC and the Rolandic operculum brain regions are responsible for generating movement patterns. These regions have weaker connections with the ACC and AI (Del Vecchio et al. 2024), suggesting that cortical structures may decode movement features more in emotional contagion than emotions themselves. In addition, IPL damage selectively affects the mapping from movement features to intention but does not impact the overall perception of movement features (Patri et al. 2020). In motor tasks, IPL activation intensity is higher than that of the IFG, and the peak of activation occurs earlier, supporting the role of the IPL as a ‘movement detector’ (Perry et al. 2018). Krautheim et al. (2020) further found that facial emotions and non-emotional motor tasks share some movement networks, with non-emotional facial movements (e.g. lip protrusion) activating the superior temporal cortex, temporo-occipital junction, postcentral gyrus, and left fusiform gyrus. This finding is consistent with our results.

The function of the IFG is quite complex and has been shown to perform different functions in experiments. For example, coupling with the dorsomedial prefrontal cortex facilitates motor synchronization (Marton-Alper et al. 2023), while its interaction with the cerebellum may also contribute to emotional processing (Sato et al. 2019, Krautheim et al. 2020). When observing facial expressions of pain, the activation intensity of the IFG is higher than that of neutral expressions (Sato et al. 2019, Li et al. 2021a, 2021b). The facial region of the IFG (IFG-FA) specifically encodes the intensity of facial expressions, while STS, ITG, and LO regions encode the category and intensity of facial expressions (Muukkonen and Salmela 2022). Coupling between the IFG and dorsolateral prefrontal cortex can predict pain relief during empathic interactions (Avnor et al. 2025). As the homologous region of human F5 neurons, the IFG may play a core role in the integration of movement and emotion (the specific functions of BA44 and BA45 are further discussed in ‘Mechanisms of emotional mimicry and emotional contagion’ section).

In addition to the frontoparietal network, emotional contagion tasks also activate the limbic system, encompassing the cingulate cortex, insula, thalamus, cerebellar cortex, and hippocampal formation. The insula, interconnected with the amygdala, facilitates the transformation of observed emotions into subjective experiences (Carr et al. 2003, Maier et al. 2023). The activation of the ACC and insula may constitute the neural substrate of emotional contagion (Zhou et al. 2020, Caruana 2022, Del Vecchio et al. 2024). The insula primarily integrates viscerosensory signals, decodes affective valence, and generates emotional experiences (Caruana 2019, Zhang et al. 2019). The ACC modulates emotional expression through event-specific ensemble patterns that regulate autonomic states (Seamans and Floresco 2022) and controls motor behaviour by regulating the frontal operculum and SMA (Monosov et al. 2020, Caruana 2022, Takeuchi et al. 2022). Furthermore, STG (BA38), as part of the extended limbic system, may integrate complex perceptual inputs with visceral emotional responses through channel-specific mechanisms (Sonkusare et al. 2020), while the thalamus filters or amplifies visual information during emotional contagion processes (Carretié et al. 2021).

Recent studies by Cuccio and Caruana (2023) and Del Vecchio et al. (2024) have demonstrated that motor resonance and emotional resonance rely on distinct neural systems. Motor resonance primarily involves the Rolandic operculum, IFG, and vPMC, which are responsible for eliciting sensorimotor responses in the face (Caruana 2022). In contrast, emotional resonance predominantly depends on the limbic system, closely associated with interoception and emotional experience (Carr et al. 2003, Ferrari et al. 2017, Caruana 2019, Liu et al. 2021, Xu et al. 2021, Del Vecchio et al. 2024). Ferrari et al. (2017) proposed a facial pathway within the emotion-related MNS, which involves key structures including the ACC, insula, and amygdala. The insular cortex may serve as a neural interface mediating between motor representation and the emotional limbic system.

According to embodied simulation theory, the MNS may also contribute to emotional state generation. Highly empathic individuals show stronger MNS activation (Hamada et al. 2022), with activation levels positively correlating with sensorimotor regions, including the IPL and IFG (Schurz et al. 2020, Plata-Bello et al. 2023). The PreG may participate in holistic emotion recognition (Sabatinelli et al. 2011, van den Berg et al. 2021). Meta-analysis indicates both PreG and IPL activation during negative emotional empathy responses (Timmers et al. 2018). Charidza and Gillmeister (2022) found happy dynamic facial expressions elicited stronger central ERD than sad and fearful expressions, with attenuation in high trait anxiety and ASD individuals, supporting motor and simulation mechanisms in emotional contagion. The MNS may facilitate motor-visual information integration and transmission to other networks for further processing (Khalil et al. 2018).

In summary, emotional contagion primarily relies on the limbic system and involves multiple interacting brain regions. The MNS likely plays a synergistic rather than dominant role in this process (Bekkali 2019, Caruana 2019, Bekkali et al. 2021). For instance, Del Vecchio et al. (2024) identified weak connectivity between the ACC/AI and VPMC/Rolandic operculum, while Caruana (2022) demonstrated that the VPMC and Rolandic operculum generate motor patterns without emotional valence. However, meta-analytic evidence has yet to establish causal relationships between cortical mirror regions and affective synchrony or experience. Activation in the IFG, SFG, and MFG may merely reflect unexecuted motor copies used for simulating emotional states (Hess 2021).

### Mechanisms of emotional mimicry and emotional contagion

In emotional contagion tasks, significant activation has been observed in the IFG (BA47/BA45/BA44), MFG, SFG, MTG, and parahippocampal gyrus, indicating specialized roles of these regions in emotional processing, with activation patterns independent of mimicking behaviour. Conversely, facial mimicry tasks elicit stronger activation in the PreG, STG, insula, and MFG. Although emotional contagion and facial mimicry involve distinct neural mechanisms, they exhibit overlapping activation in several regions, including the IFG, PreG, STG, insula, and cingulate gyrus, suggesting shared neural functions in certain aspects.

The embodied emotion model posits that individuals can elicit corresponding emotional experiences by simulating facial expressions they would produce in similar situations (Gallese et al. 2004, Decety et al. 2008, Ding et al. 2020, Zuberer et al. 2022). Neuroimaging studies have further demonstrated the functional correlation between emotional contagion and facial mimicry at the neural level (Hirsch et al. 2023). However, Hess and Blairy’s (2001) research indicates that facial muscle mimicry during dynamic facial expression observation does not directly predict emotional experience.

Based on our results, facial mimicry does not appear to be part of the neural system involved in emotional contagion. In emotional contagion tasks, regions such as the IFG (BA47), SFG, and MTG show stronger activation. Even though SFG is not traditionally classified within the limbic system, studies suggest that it is related to emotion recognition and regulation (Kennedy et al. 2021, Li et al. 2021a, 2021b). The MTG plays an important role in the recognition of facial expressions and the formation of emotional memories (Ding et al. 2020, Zuberer et al. 2022). Activation of the insula is more robust in emotional contagion and shows a leftward lateralization (Dai et al. 2018). Compared to the right insula, the left insula, as part of the bilateral frontotemporal limbic network, is involved in emotion recognition of facial expressions (Holtmann et al. 2022) and seems to be particularly important for processing negative facial expressions (Holtmann et al. 2020). The parahippocampal gyrus also shows strong activation during the encoding and retrieval of emotional contextual memory (Dahlgren et al. 2020).

In contrast to emotional contagion, facial mimicry primarily relies on the motor planning and execution functions of the PreG (Bello et al. 2020), which precisely controls facial muscle movements to imitate observed expressions (Krane et al. 2022). The MNS components involved in mimicry behaviour—including the IFG, IPL, insula, and SMA—are closely associated with spontaneous facial muscle responses (Rymarczyk et al. 2019). Insula activation during facial mimicry may reflect changes in emotional experience, even when such experiences remain non-conscious (Nguyen et al. 2016, Evrard 2019, Di Cesare et al. 2020). The temporal pole and extended insula may play distinct roles in facial mimicry, while the STG facilitates comparison between observed actions and their sensory consequences (Cattaneo et al. 2010, Kilintari et al. 2014).

Comparative analysis of emotional contagion and facial mimicry reveals distinct IFG activation patterns. During emotional contagion tasks, significant activation is observed in the left IFGorb (BA47) and right IFGtri (BA45). The IFGorb (BA47) forms an emotion regulation network with the ACC, insula, and amygdala, playing a crucial role in emotional processing and intrinsic mimicry response modulation (Lee et al. 2008, Belyk et al. 2017). As a convergence zone between dorsal and ventral streams, the IFGtri (BA45) likely contributes to action understanding in emotional contagion through integration of motor and visual information (Hamzei et al. 2016, Correia et al. 2019).

In facial mimicry tasks, the IFGope (BA44) demonstrates greater activation, supporting action simulation processes and coordinating with parietal, motor, and somatosensory cortices (Zarka et al. 2022). The IFGope’s (BA44) pivotal role in the MNS has been empirically validated, with electrical stimulation of this region eliciting facial muscle movements (Del Vecchio et al. 2024). It co-activates with the ACC, anterior insula, and SMA to coordinate facial actions and emotional resonance (Jabbi and Keysers 2008, Wang et al. 2024).

Although these processes share overlapping neural mechanisms, changes in facial expressions do not always directly elicit subjective emotional experiences, nor does emotional synchrony necessarily manifest in overt facial expressions. The MNS provides a neural foundation for both emotional contagion and facial mimicry by mediating sensorimotor information processing.

### Functional connectivity in conjunction regions

Our finding suggested that the cingulate gyrus, MFG, and IPL are shared regions between the two behavioural domains.

These shared regions form complex connectivity networks with the thalamus, lentiform nucleus, PreG, and insula, participating in emotional integration, motor control, and affective valence processing. According to Kim et al. (2020), the PreG–MFG connectivity plays a crucial role in motor networks, consistent with our findings of their co-activation during both emotional contagion and mimicry processes. Furthermore, activation patterns in the thalamus and lentiform nucleus during emotional contagion tasks resemble those observed in emotion recognition tasks (Li et al. 2010, Ciumas et al. 2017), suggesting their significant contribution to emotional information processing.

The insula also plays a pivotal role. Our results demonstrate that the insula engages the IPL and MFG within the action observation network, consistent with findings from Zhang et al. (2022) and Grosbras and Paus (2006). The insula not only decodes bodily signals but also transforms them into emotional experiences. Thus, it may serve as a neural interface in emotional contagion and mimicry mechanisms, integrating emotional and motor information.

Moreover, shared activation regions between emotional contagion and facial mimicry exhibit hemispheric asymmetry. Although these overlapping regions are distributed across both hemispheres, co-activation patterns predominantly occur in the right hemisphere, potentially indicating its dominant role in emotional processing. Specifically, right hemispheric regions may be critically involved in the synchrony mechanisms between emotion and motion during emotional contagion tasks.

## Limitations

The functional connectivity analysis in this study was conducted using data pooled from various cognitive paradigms. While this approach provides valuable supplementary information for meta-analysis, it presents certain limitations. By aggregating data from different tasks and experimental conditions, we might obscure crucial differences in brain activation patterns, particularly regarding region-specific processing sensitivity. The statistical power of regional activation detection may be compromised by emotional expression, as random-effects fMRI experiments and whole-brain analyses exhibit relative insensitivity in this regard. Future studies could employ Bayesian regression and multivariate pattern analysis to enhance sensitivity to emotion-specific processing.

Second, although the MNS and emotional systems operate somewhat independently, the process of emotional sharing requires coordinated activity across multiple brain regions. Future research should focus on elucidating the specific role and extent of MNS involvement in emotional sharing, representing a promising direction for further exploration. Additionally, as our meta-analysis did not incorporate participants’ subjective emotional data, subsequent studies should integrate behavioural measures with neuroimaging data to establish clearer relationships between brain activation and emotional contagion.

Finally, to mitigate potential publication bias in the current meta-analysis, future research could enhance the comprehensiveness and robustness of analyses by acquiring and integrating unpublished data, thereby expanding the sample scope.

## Conclusion

Although emotional contagion and facial mimicry exhibit distinct neural mechanisms, they share multiple brain regions, including the cingulate gyrus, MFG, and IPL, which collectively form a complex neural network interacting with key nodes such as the thalamus, lentiform nucleus, insula, and PreG. While the MNS and emotional systems operate independently in certain aspects, emotional contagion relies on the limbic system and involves coordinated activity across multiple brain regions, with the MNS playing a supportive rather than dominant role. Furthermore, the brain connectivity networks of both processes reveal resonance mechanisms between emotion and motion, reflecting synchronization of emotional experience and sensorimotor perception, indicating functional overlap and synergistic interactions in specific brain regions.

## Supplementary Material

nsaf091_Supplementary_Data

## Data Availability

This manuscript is a meta-analysis paper that does not include any primary research involving data and code. Data and code would have to be requested from the primary sources that are cited in the current manuscript and supplementary material.

## References

[nsaf091-B1] Acar F , Seurinck R, Eickhoff SB et al Assessing robustness against potential publication bias in activation likelihood estimation (ALE) meta-analyses for fMRI. PLoS One 2018;13:e0208177. 10.1371/journal.pone.0208177 30500854 PMC6267999

[nsaf091-B2] Aviezer H , Ensenberg N, Hassin RR. The inherently contextualized nature of facial emotion perception. Curr Opin Psychol 2017;17:47–54. 10.1016/j.copsyc.2017.06.006 28950972

[nsaf091-B3] Avnor Y , Atias D, Markus A et al It takes two to empathize: interbrain coupling contributes to distress regulation. Emotion 2025;25:736–54. 10.1037/emo0001431 39531679

[nsaf091-B4] Barrett LF , Adolphs R, Marsella S et al Emotional expressions reconsidered: challenges to inferring emotion from human facial movements. Psychol Sci Public Interest 2019;20:1–68. 10.1177/1529100619832930 31313636 PMC6640856

[nsaf091-B5] Bastiaansen JA , Thioux M, Keysers C. Evidence for mirror systems in emotions. Philos Trans R Soc Lond B Biol Sci 2009;364:2391–404. 10.1098/rstb.2009.0058 19620110 PMC2865077

[nsaf091-B6] Bekkali S. The human mirror neuron system and empathy: a multimodal investigation, Deakin University, 2019.

[nsaf091-B7] Bekkali S , Youssef GJ, Donaldson PH et al Is the putative mirror neuron system associated with empathy? A systematic review and meta-analysis. Neuropsychol Rev 2021;31:14–57. 10.1007/s11065-020-09452-6 32876854

[nsaf091-B8] Bello UM , Kranz GS, Winser SJ et al Neural processes underlying mirror-induced visual illusion: an activation likelihood estimation meta-analysis. Front Hum Neurosci 2020;14:276. 10.3389/fnhum.2020.00276 32848663 PMC7412952

[nsaf091-B9] Belyk M , Brown S, Lim J et al Convergence of semantics and emotional expression within the IFG pars orbitalis. Neuroimage 2017;156:240–8. 10.1016/j.neuroimage.2017.04.020 28400265

[nsaf091-B10] Binder E , Dovern A, Hesse MD et al Lesion evidence for a human mirror neuron system. Cortex 2017;90:125–37. 10.1016/j.cortex.2017.02.008 28391066

[nsaf091-B11] Braadbaart L , de Grauw H, Perrett DI et al The shared neural basis of empathy and facial imitation accuracy. Neuroimage 2014;84:367–75. 10.1016/j.neuroimage.2013.08.061 24012546

[nsaf091-B12] Budell L , Kunz M, Jackson PL et al Mirroring pain in the brain: emotional expression versus motor imitation. PLoS One 2015;10:e0107526. 10.1371/journal.pone.0107526 25671563 PMC4324963

[nsaf091-B13] Carr L , Iacoboni M, Dubeau MC et al Neural mechanisms of empathy in humans: a relay from neural systems for imitation to limbic areas. Proc Natl Acad Sci U S A 2003;100:5497–502. 10.1073/pnas.0935845100 12682281 PMC154373

[nsaf091-B14] Carretié L , Yadav RK, Méndez-Bértolo C. The missing link in early emotional processing. Emotion Rev 2021;13:225–44. 10.1177/17540739211022821

[nsaf091-B15] Caruana F. The integration of emotional expression and experience: a pragmatist review of recent evidence from brain stimulation. Emotion Rev 2019;11:27–38. 10.1177/1754073917723461

[nsaf091-B16] Caruana F. Two simulation systems in the human frontal cortex? Disentangling between motor simulation and emotional mirroring using laughter. Cortex 2022;148:215–7. 10.1016/j.cortex.2021.09.011 34696898

[nsaf091-B17] Cattaneo L , Sandrini M, Schwarzbach J. State-dependent TMS reveals a hierarchical representation of observed acts in the temporal, parietal, and premotor cortices. Cereb Cortex 2010;20:2252–8. 10.1093/cercor/bhp291 20051360

[nsaf091-B18] Cerniglia L , Bartolomeo L, Capobianco M et al Intersections and divergences between empathizing and mentalizing: development, recent advancements by neuroimaging and the future of animal modeling. Front Behav Neurosci 2019;13:212. 10.3389/fnbeh.2019.00212 31572143 PMC6754072

[nsaf091-B19] Charidza CA , Gillmeister H. Differential beta desynchronisation responses to dynamic emotional facial expressions are attenuated in higher trait anxiety and autism. Cogn Affect Behav Neurosci 2022;22:1404–20. 10.3758/s13415-022-01015-x 35761029 PMC9622532

[nsaf091-B20] Ciumas C , Laurent A, Saignavongs M et al Behavioral and fMRI responses to fearful faces are altered in benign childhood epilepsy with centrotemporal spikes (BCECTS). Epilepsia 2017;58:1716–27. 10.1111/epi.13858 28762475

[nsaf091-B21] Correia AI , Branco P, Martins M et al Resting-state connectivity reveals a role for sensorimotor systems in vocal emotional processing in children. Neuroimage 2019;201:116052. 10.1016/j.neuroimage.2019.116052 31351162

[nsaf091-B22] Cuccio V , Caruana F. Motor simulation of facial expressions, but not emotional mirroring, depends on automatic sensorimotor abduction. In: Magnani L (ed.), Handbook of Abductive Cognition. Cham: Springer International Publishing, 2023, 1709–26.

[nsaf091-B23] Dahlgren K , Ferris C, Hamann S. Neural correlates of successful emotional episodic encoding and retrieval: an SDM meta-analysis of neuroimaging studies. Neuropsychologia 2020;143:107495. 10.1016/j.neuropsychologia.2020.107495 32416099

[nsaf091-B24] Dai YJ , Zhang X, Yang Y et al Gender differences in functional connectivities between insular subdivisions and selective pain-related brain structures. J Headache Pain 2018;19:24. 10.1186/s10194-018-0849-z 29541875 PMC5852124

[nsaf091-B25] Danish JA , Enyedy N, Saleh A et al Learning in embodied activity framework: a sociocultural framework for embodied cognition. Intern J Comput-Support Collab Learn 2020;15:49–87. 10.1007/s11412-020-09317-3

[nsaf091-B26] De Vignemont F , Singer T. The empathic brain: how, when and why? Trends Cogn Sci 2006;10:435–41. 10.1016/j.tics.2006.08.008 16949331

[nsaf091-B27] De Waal FB. The antiquity of empathy. Science 2012;336:874–6. 10.1126/science.1220999 22605767

[nsaf091-B28] Decety J , Michalska KJ, Akitsuki Y. Who caused the pain? An fMRI investigation of empathy and intentionality in children. Neuropsychologia 2008;46:2607–14. 10.1016/j.neuropsychologia.2008.05.026 18573266

[nsaf091-B29] Del Vecchio M , Avanzini P, Gerbella M et al Anatomo-functional basis of emotional and motor resonance elicited by facial expressions. Brain 2024;147:3018–31. 10.1093/brain/awae050 38365267 PMC12007602

[nsaf091-B30] Di Cesare G , Gerbella M, Rizzolatti G. The neural bases of vitality forms. Natl Sci Rev 2020;7:202–13. 10.1093/nsr/nwz187 34692032 PMC8288905

[nsaf091-B31] Ding J , Wang Y, Wang C et al Negative impact of sadness on response inhibition in females: an explicit emotional stop signal task fMRI study. Front Behav Neurosci 2020;14:119. 10.3389/fnbeh.2020.00119 32903296 PMC7396530

[nsaf091-B32] Downey JA. Hierarchy and happiness: the influence of emotion on administrative job satisfaction. Community Coll J Res Pract 2008;32:597–606. 10.1080/10668920600859996

[nsaf091-B33] Eickhoff SB , Bzdok D, Laird AR et al Co-activation patterns distinguish cortical modules, their connectivity and functional differentiation. Neuroimage 2011;57:938–49. 10.1016/j.neuroimage.2011.05.021 21609770 PMC3129435

[nsaf091-B34] Eickhoff SB , Laird AR, Grefkes C et al Coordinate-based activation likelihood estimation meta-analysis of neuroimaging data: a random-effects approach based on empirical estimates of spatial uncertainty. Hum Brain Mapp 2009;30:2907–26. 10.1002/hbm.20718 19172646 PMC2872071

[nsaf091-B35] Eickhoff SB , Nichols TE, Laird AR et al Behavior, sensitivity, and power of activation likelihood estimation characterized by massive empirical simulation. Neuroimage 2016;137:70–85. 10.1016/j.neuroimage.2016.04.072 27179606 PMC4981641

[nsaf091-B36] Errante A , Ziccarelli S, Mingolla GP et al Decoding grip type and action goal during the observation of reaching-grasping actions: a multivariate fMRI study. Neuroimage 2021;243:118511. 10.1016/j.neuroimage.2021.118511 34450263

[nsaf091-B37] Evrard HC. The organization of the primate insular cortex. Front Neuroanat 2019;13:43. 10.3389/fnana.2019.00043 31133822 PMC6517547

[nsaf091-B38] Farina M. Embodied cognition: dimensions, domains and applications. Adapt Behav 2021;29:73–88. 10.1177/1059712320912963

[nsaf091-B40] Ferrari PF , Gerbella M, Coude G et al Two different mirror neuron networks: the sensorimotor (hand) and limbic (face) pathways. Neuroscience 2017;358:300–15. 10.1016/j.neuroscience.2017.06.052 28687313 PMC6063080

[nsaf091-B41] Fischer A , Hess U. Mimicking emotions. Curr Opin Psychol 2017;17:151–5. 10.1016/j.copsyc.2017.07.008 28950963

[nsaf091-B42] Franklin Z. Emotional contagion: how we mimic the emotions of those similar to us. Berkeley Sci J 2019;24:18–20. 10.5070/BS3241046897

[nsaf091-B43] Gallese V. Mirror neurons, embodied simulation, and the neural basis of social identification. Psychoanal Dialogues 2009;19:519–36. 10.1080/10481880903231910

[nsaf091-B44] Gallese V , Keysers C, Rizzolatti G. A unifying view of the basis of social cognition. Trends Cogn Sci 2004;8:396–403. 10.1016/j.tics.2004.07.002 15350240

[nsaf091-B45] Goldman A , de Vignemont F. Is social cognition embodied? Trends Cogn Sci 2009;13:154–9. 10.1016/j.tics.2009.01.007 19269881

[nsaf091-B46] Grosbras MH , Paus T. Brain networks involved in viewing angry hands or faces. Cereb Cortex 2006;16:1087–96. 10.1093/cercor/bhj050 16221928

[nsaf091-B47] Hamada M , Matsubayashi J, Tanaka K et al People with high empathy show increased cortical activity around the left medial parieto-occipital sulcus after watching social interaction of on-screen characters. Cereb Cortex 2022;32:3581–601. 10.1093/cercor/bhab435 35059713

[nsaf091-B48] Hamzei F , Vry MS, Saur D et al The dual-loop model and the human mirror neuron system: an exploratory combined fMRI and DTI study of the inferior frontal gyrus. Cereb Cortex 2016;26:2215–24. 10.1093/cercor/bhv066 25828568

[nsaf091-B49] Hatfield E , Cacioppo JT, Rapson RL. Emotional contagion. Curr Dir Psychol Sci 1993;2:96–100. 10.1111/1467-8721.ep10770953

[nsaf091-B50] Hennenlotter A , Schroeder U, Erhard P et al A common neural basis for receptive and expressive communication of pleasant facial affect. Neuroimage 2005;26:581–91. 10.1016/j.neuroimage.2005.01.057 15907315

[nsaf091-B51] Hess U. Who to whom and why: the social nature of emotional mimicry. Psychophysiology 2021;58:e13675. 10.1111/psyp.13675 32915999

[nsaf091-B52] Hess U , Blairy S. Facial mimicry and emotional contagion to dynamic emotional facial expressions and their influence on decoding accuracy. Int J Psychophysiol 2001;40:129–41. 10.1016/s0167-8760(00)00161-6 11165351

[nsaf091-B53] Hickok G. Eight problems for the mirror neuron theory of action understanding in monkeys and humans. J Cogn Neurosci 2009;21:1229–43. 10.1162/jocn.2009.21189 19199415 PMC2773693

[nsaf091-B54] Hirsch J , Zhang X, Noah JA et al Neural mechanisms for emotional contagion and spontaneous mimicry of live facial expressions. Philos Trans R Soc Lond B Biol Sci 2023;378:20210472. 10.1098/rstb.2021.0472 36871593 PMC9985973

[nsaf091-B55] Holtmann O , Bruchmann M, Monig C et al Lateralized deficits of disgust processing after insula-basal ganglia damage. Front Psychol 2020;11:1429. 10.3389/fpsyg.2020.01429 32714249 PMC7347022

[nsaf091-B56] Holtmann O , Franz M, Monig C et al Lateralized deficits in arousal processing after insula lesions: behavioral and autonomic evidence. Cortex 2022;148:168–79. 10.1016/j.cortex.2021.12.013 35180480

[nsaf091-B57] Jabbi M , Keysers C. Inferior frontal gyrus activity triggers anterior insula response to emotional facial expressions. Emotion 2008;8:775–80. 10.1037/a0014194 19102588

[nsaf091-B58] Kennedy M , Simcock G, Jamieson D et al Elucidating the neural correlates of emotion recognition in children with sub-clinical anxiety. J Psychiatr Res 2021;143:75–83. 10.1016/j.jpsychires.2021.08.024 34461352

[nsaf091-B59] Keysers C , Gazzola V. Hebbian learning and predictive mirror neurons for actions, sensations and emotions. Philos Trans R Soc Lond B Biol Sci 2014;369:20130175. 10.1098/rstb.2013.0175 24778372 PMC4006178

[nsaf091-B60] Keysers C , Gazzola V. Vicarious emotions of fear and pain in rodents. Affect Sci 2023;4:662–71. 10.1007/s42761-023-00198-x 38156261 PMC10751282

[nsaf091-B61] Keysers C , Paracampo R, Gazzola V. What neuromodulation and lesion studies tell us about the function of the mirror neuron system and embodied cognition. Curr Opin Psychol 2018;24:35–40. 10.1016/j.copsyc.2018.04.001 29734039 PMC6173305

[nsaf091-B62] Khalil R , Tindle R, Boraud T et al Social decision making in autism: on the impact of mirror neurons, motor control, and imitative behaviors. CNS Neurosci Ther 2018;24:669–76. 10.1111/cns.13001 29963752 PMC6055683

[nsaf091-B63] Kilintari M , Raos V, Savaki HE. Involvement of the superior temporal cortex in action execution and action observation. J Neurosci 2014;34:8999–9011. 10.1523/JNEUROSCI.0736-14.2014 24990920 PMC6608254

[nsaf091-B64] Kim EJ , Son JW, Park SK et al Cognitive and emotional empathy in young adolescents: an fMRI study. Soa Chongsonyon Chongsin Uihak 2020;31:121–30. 10.5765/jkacap.200020 32665756 PMC7350548

[nsaf091-B65] Krane NA , Loyo M, Pollock J et al Exploratory study of the brain response in facial synkinesis after bell palsy with systematic review and meta-analysis of the literature. AJNR Am J Neuroradiol 2022;43:1470–5. 10.3174/ajnr.A7619 36574328 PMC9575525

[nsaf091-B66] Krautheim JT , Steines M, Dannlowski U et al Emotion specific neural activation for the production and perception of facial expressions. Cortex 2020;127:17–28. 10.1016/j.cortex.2020.01.026 32155474

[nsaf091-B67] Laird AR , Eickhoff SB, Kurth F et al Ale meta-analysis workflows via the brainmap database: progress towards a probabilistic functional brain atlas. Front Neuroinform 2009;3:23. 10.3389/neuro.11.023.2009 19636392 PMC2715269

[nsaf091-B68] Lancaster JL , Tordesillas-Gutierrez D, Martinez M et al Bias between MNI and Talairach coordinates analyzed using the ICBM-152 brain template. Hum Brain Mapp 2007;28:1194–205. 10.1002/hbm.20345 17266101 PMC6871323

[nsaf091-B69] Langner R , Rottschy C, Laird AR et al Meta-analytic connectivity modeling revisited: controlling for activation base rates. Neuroimage 2014;99:559–70. 10.1016/j.neuroimage.2014.06.007 24945668 PMC4112007

[nsaf091-B70] Lee TW , Dolan RJ, Critchley HD. Controlling emotional expression: behavioral and neural correlates of nonimitative emotional responses. Cereb Cortex 2008;18:104–13. 10.1093/cercor/bhm035 17483530 PMC2275800

[nsaf091-B71] Li H , Chan RC, McAlonan GM et al Facial emotion processing in schizophrenia: a meta-analysis of functional neuroimaging data. Schizophr Bull 2010;36:1029–39. 10.1093/schbul/sbn190 19336391 PMC2930350

[nsaf091-B72] Li W , Yang P, Ngetich RK et al Differential involvement of frontoparietal network and insula cortex in emotion regulation. Neuropsychologia 2021a;161:107991. 10.1016/j.neuropsychologia.2021.107991 34391808

[nsaf091-B73] Li X , Krol MA, Jahani S et al Brain correlates of motor complexity during observed and executed actions. Sci Rep 2020;10:10965. 10.1038/s41598-020-67327-5 32620887 PMC7335074

[nsaf091-B74] Li Y , Li W, Zhang T et al Probing the role of the right inferior frontal gyrus during pain-related empathy processing: evidence from fMRI and TMS. Hum Brain Mapp 2021b;42:1518–31. 10.1002/hbm.25310 33283946 PMC7927301

[nsaf091-B75] Liu M , Liu CH, Zheng S et al Reexamining the neural network involved in perception of facial expression: a meta-analysis. Neurosci Biobehav Rev 2021;131:179–91. 10.1016/j.neubiorev.2021.09.024 34536463

[nsaf091-B76] Maier F , Greuel A, Hoock M et al Impaired self-awareness of cognitive deficits in Parkinson’s disease relates to cingulate cortex dysfunction. Psychol Med 2023;53:1244–53. 10.1017/S0033291721002725 37010224 PMC10009405

[nsaf091-B77] Iacoboni M. Face to face: the neural basis of social mirroring and empathy. Psychiatr Ann 2007;37:236–41.

[nsaf091-B78] Marton-Alper IZ , Markus A, Nevat M et al Differential contribution of between and within-brain coupling to movement synchronization. Hum Brain Mapp 2023;44:4136–51. 10.1002/hbm.26335 37195028 PMC10258530

[nsaf091-B79] Mazza M , Tempesta D, Pino MC et al Neural activity related to cognitive and emotional empathy in post-traumatic stress disorder. Behav Brain Res 2015;282:37–45. 10.1016/j.bbr.2014.12.049 25555525

[nsaf091-B80] Monosov IE , Haber SN, Leuthardt EC et al Anterior cingulate cortex and the control of dynamic behavior in primates. Curr Biol 2020;30:R1442–54. 10.1016/j.cub.2020.10.009 33290716 PMC8197026

[nsaf091-B81] Müller VI , Cieslik EC, Laird AR et al Ten simple rules for neuroimaging meta-analysis. Neurosci Biobehav Rev 2018;84:151–61. 10.1016/j.neubiorev.2017.11.012 29180258 PMC5918306

[nsaf091-B82] Muukkonen I , Salmela VR. Representational structure of fMRI/EEG responses to dynamic facial expressions. Neuroimage 2022;263:119631. 10.1016/j.neuroimage.2022.119631 36113736

[nsaf091-B83] Nguyen VT , Breakspear M, Hu X et al The integration of the internal and external milieu in the insula during dynamic emotional experiences. Neuroimage 2016;124:455–63. 10.1016/j.neuroimage.2015.08.078 26375211

[nsaf091-B84] Nummenmaa L , Hirvonen J, Parkkola R et al Is emotional contagion special? An fMRI study on neural systems for affective and cognitive empathy. Neuroimage 2008;43:571–80. 10.1016/j.neuroimage.2008.08.014 18790065

[nsaf091-B85] Olszanowski M , Lewandowska P, Ozimek A et al The effect of facial self-resemblance on emotional mimicry. J Nonverbal Behav 2022;46:197–213. 10.1007/s10919-021-00395-x

[nsaf091-B86] Olszanowski M , Wrobel M, Hess U. Mimicking and sharing emotions: a re-examination of the link between facial mimicry and emotional contagion. Cogn Emot 2020;34:367–76. 10.1080/02699931.2019.1611543 31072246

[nsaf091-B87] Palagi E , Celeghin A, Tamietto M et al The neuroethology of spontaneous mimicry and emotional contagion in human and non-human animals. Neurosci Biobehav Rev 2020;111:149–65. 10.1016/j.neubiorev.2020.01.020 31972204

[nsaf091-B88] Patri JF , Cavallo A, Pullar K et al Transient disruption of the inferior parietal lobule impairs the ability to attribute intention to action. Curr Biol 2020;30:4594–605 e7. 10.1016/j.cub.2020.08.104 32976808 PMC7726027

[nsaf091-B89] Paz LV , Viola TW, Milanesi BB et al Contagious depression: automatic mimicry and the mirror neuron system—a review. Neurosci Biobehav Rev 2022;134:104509. 10.1016/j.neubiorev.2021.12.032 34968526

[nsaf091-B90] Perry A , Stiso J, Chang EF et al Mirroring in the human brain: deciphering the spatial-temporal patterns of the human mirror neuron system. Cereb Cortex 2018;28:1039–48. 10.1093/cercor/bhx013 28137724 PMC6059139

[nsaf091-B91] Plata-Bello J , Privato N, Modrono C et al Empathy modulates the activity of the sensorimotor mirror neuron system during pain observation. Behav Sci (Basel) 2023;13:947. 10.3390/bs13110947 PMC10669321 37998694

[nsaf091-B92] Preston SD , de Waal FB. Empathy: its ultimate and proximate bases. Behav Brain Sci 2002;25:1–20; discussion 20–71. 10.1017/s0140525x02000018 12625087

[nsaf091-B93] Prochazkova E , Kret ME. Connecting minds and sharing emotions through mimicry: a neurocognitive model of emotional contagion. Neurosci Biobehav Rev 2017;80:99–114. 10.1016/j.neubiorev.2017.05.013 28506927

[nsaf091-B94] Rizzolatti G , Caruana F. Some considerations on de Waal and Preston review. Nat Rev Neurosci 2017;18:769. 10.1038/nrn.2017.139 29097788

[nsaf091-B95] Rizzolatti G , Sinigaglia C. The functional role of the parieto-frontal mirror circuit: interpretations and misinterpretations. Nat Rev Neurosci 2010;11:264–74. 10.1038/nrn2805 20216547

[nsaf091-B96] Rymarczyk K , Żurawski Ł, Jankowiak-Siuda K et al Empathy in facial mimicry of fear and disgust: simultaneous EMG-fMRI recordings during observation of static and dynamic facial expressions. Front Psychol 2019;10:701. 10.3389/fpsyg.2019.00701 30971997 PMC6445885

[nsaf091-B97] Sabatinelli D , Fortune EE, Li Q et al Emotional perception: meta-analyses of face and natural scene processing. Neuroimage 2011;54:2524–33. 10.1016/j.neuroimage.2010.10.011 20951215

[nsaf091-B98] Sato W , Kochiyama T, Uono S et al Widespread and lateralized social brain activity for processing dynamic facial expressions. Hum Brain Mapp 2019;40:3753–68. 10.1002/hbm.24629 31090126 PMC6865540

[nsaf091-B99] Schmidt SNL , Hass J, Kirsch P et al The human mirror neuron system-a common neural basis for social cognition? Psychophysiology 2021;58:e13781. 10.1111/psyp.13781 33576063

[nsaf091-B100] Schurz M , Maliske L, Kanske P. Cross-network interactions in social cognition: a review of findings on task related brain activation and connectivity. Cortex 2020;130:142–57. 10.1016/j.cortex.2020.05.006 32653744

[nsaf091-B101] Schurz M , Radua J, Tholen MG et al Toward a hierarchical model of social cognition: a neuroimaging meta-analysis and integrative review of empathy and theory of mind. Psychol Bull 2021;147:293–327. 10.1037/bul0000303 33151703

[nsaf091-B102] Seamans JK , Floresco SB. Event-based control of autonomic and emotional states by the anterior cingulate cortex. Neurosci Biobehav Rev 2022;133:104503. 10.1016/j.neubiorev.2021.12.026 34922986

[nsaf091-B103] Siposova B , Carpenter M. A new look at joint attention and common knowledge. Cognition 2019;189:260–74. 10.1016/j.cognition.2019.03.019 31015079

[nsaf091-B104] Sonkusare S , Nguyen VT, Moran R et al Intracranial-EEG evidence for medial temporal pole driving amygdala activity induced by multi-modal emotional stimuli. Cortex 2020;130:32–48. 10.1016/j.cortex.2020.05.018 32640373

[nsaf091-B105] Takeuchi D , Roy D, Muralidhar S et al Cingulate-motor circuits update rule representations for sequential choice decisions. Nat Commun 2022;13:4545. 10.1038/s41467-022-32142-1 35927275 PMC9352796

[nsaf091-B106] Timmers I , Park AL, Fischer MD et al Is empathy for pain unique in its neural correlates? A meta-analysis of neuroimaging studies of empathy. Front Behav Neurosci 2018;12:289. 10.3389/fnbeh.2018.00289 30542272 PMC6277791

[nsaf091-B107] Turkeltaub PE , Eden GF, Jones KM et al Meta-analysis of the functional neuroanatomy of single-word reading: method and validation. Neuroimage 2002;16:765–80. 10.1006/nimg.2002.1131 12169260

[nsaf091-B108] Van den Berg NS , de Haan EHF, Huitema RB et al; Visual Brain Group. The neural underpinnings of facial emotion recognition in ischemic stroke patients. J Neuropsychol 2021;15:516–32. 10.1111/jnp.12240 33554463 PMC8518120

[nsaf091-B109] Van der Gaag C , Minderaa RB, Keysers C. Facial expressions: what the mirror neuron system can and cannot tell us. Soc Neurosci 2007;2:179–222. 10.1080/17470910701376878 18633816

[nsaf091-B110] Van der Schalk J , Fischer A, Doosje B et al Convergent and divergent responses to emotional displays of ingroup and outgroup. Emotion 2011;11:286–98. 10.1037/a0022582 21500898

[nsaf091-B111] Wang J , Yang J, Yang Z et al Boosting interpersonal emotion regulation through facial imitation: functional neuroimaging foundations. Cereb Cortex 2024;34:bhad402. 10.1093/cercor/bhad402 37943770

[nsaf091-B112] Wicker B , Keysers C, Plailly J et al Both of us disgusted in My insula: the common neural basis of seeing and feeling disgust. Neuron 2003;40:655–64. 10.1016/s0896-6273(03)00679-2 14642287

[nsaf091-B113] Xu P , Peng S, Luo YJ et al Facial expression recognition: a meta-analytic review of theoretical models and neuroimaging evidence. Neurosci Biobehav Rev 2021;127:820–36. 10.1016/j.neubiorev.2021.05.023 34052280

[nsaf091-B114] Zarka D , Cebolla AM, Cheron G. Mirror neurons, neural substrate of action understanding? Encephale 2022;48:83–91. 10.1016/j.encep.2021.06.005 34625217

[nsaf091-B115] Zhang S , Zhang Y, Ma W et al Neural correlates of negative emotion processing in subthreshold depression. Soc Cogn Affect Neurosci 2022;17:655–61. 10.1093/scan/nsac003 35156124 PMC9250298

[nsaf091-B116] Zhang Y , Zhou W, Wang S et al The roles of subdivisions of human insula in emotion perception and auditory processing. Cereb Cortex 2019;29:517–28. 10.1093/cercor/bhx334 29342237

[nsaf091-B117] Zhou F , Li J, Zhao W et al Empathic pain evoked by sensory and emotional-communicative cues share common and process-specific neural representations. *eLife*, *9*, e56929. 10.7554/eLife.56929 PMC7505665 32894226

[nsaf091-B118] Zuberer A , Schwarz L, Kreifelts B et al Neural basis of impaired emotion recognition in adult attention-deficit/hyperactivity disorder. Biol Psychiatry Cogn Neurosci Neuroimaging 2022;7:680–7. 10.1016/j.bpsc.2020.11.013 33551283

