# Supplementary material for: The shared neural substrates of emotional mimicry and emotional contagion: an activation likelihood estimation meta-analysis and meta-analytic connectivity modeling analysis
Source: Soc Cogn Affect Neurosci. 2025 Sep 10;20(1):nsaf091. doi: 10.1093/scan/nsaf091 (PMC12542505; doi:10.1093/scan/nsaf091)
Supplement: nsaf091_Supplementary_Data [file nsaf091_supplementary_data.zip › scan-24-149-File015.docx]

Supplementary Materials

Supplementary Tables

Supplementary Table 3

MACM results: areas of functional coactivation associated with cluster 2(Right cerebrum: middle frontal gyrus).

| Cluster | Volume | Hemisphere | Region | BA(s) | Coordinate | | | ALE |
| --- | --- | --- | --- | --- | --- | --- | --- | --- |
|  | (mm^3^) |  |  |  | x | y | z | （×10^-2^） |
| 1 | 222896 | Right | Medial Frontal Gyrus | 6 | 6 | 8 | 60 | 22.35 |
|  |  | Right | Insula |  | 40 | 18 | -2 | 8.84 |
|  |  | Left | Insula | 13 | -34 | 22 | 2 | 8.09 |
|  |  | Right | Cingulate Gyrus | 32 | 8 | 20 | 36 | 7.14 |
|  |  | Right | Middle Frontal Gyrus | 6 | 46 | 4 | 46 | 6.99 |
|  |  | Left | Insula | 13 | -50 | 10 | 0 | 5.80 |
|  |  | Left | Insula | 13 | -48 | 14 | -2 | 5.78 |
|  |  | Left | Lentiform Nucleus |  | -20 | 6 | 6 | 5.77 |
|  |  | Right | Lentiform Nucleus |  | 22 | 4 | 4 | 5.73 |
|  |  | Right | Inferior Frontal Gyrus | 9 | 48 | 14 | 18 | 5.56 |
|  |  | Left | Precuneus | 7 | -10 | -68 | 52 | 5.55 |
|  |  | Left | Inferior Frontal Gyrus | 9 | -46 | 10 | 26 | 5.43 |
|  |  | Right | Middle Frontal Gyrus | 6 | 40 | 0 | 52 | 5.33 |
|  |  | Left | Precentral Gyrus | 6 | -44 | 2 | 46 | 5.31 |
|  |  | Left | Superior Parietal Lobule | 7 | -26 | -64 | 48 | 5.28 |
|  |  | Left | Precentral Gyrus | 6 | -50 | 8 | 34 | 5.23 |
|  |  | Right | Postcentral Gyrus | 40 | 62 | -22 | 22 | 5.20 |
|  |  | Left | Precentral Gyrus | 6 | -44 | -4 | 46 | 5.19 |
|  |  | Right | Thalamus |  | 10 | -14 | 4 | 5.16 |
|  |  | Right | Precuneus | 7 | 20 | -66 | 58 | 5.12 |
|  |  | Left | Precentral Gyrus | 6 | -48 | 2 | 38 | 5.05 |
|  |  | Left | Thalamus |  | -12 | -12 | 2 | 4.99 |
|  |  | Left | Inferior Parietal Lobule | 40 | -34 | -50 | 44 | 4.96 |
|  |  | Left | Inferior Parietal Lobule | 40 | -38 | -40 | 48 | 4.87 |
|  |  | Right | Inferior Parietal Lobule | 40 | 60 | -36 | 42 | 4.83 |
|  |  | Left | Middle Frontal Gyrus | 46 | -44 | 34 | 18 | 4.82 |
|  |  | Left | Precuneus | 7 | -20 | -62 | 56 | 4.73 |
|  |  | Left | Precuneus | 7 | -30 | -50 | 54 | 4.72 |
|  |  | Left | Inferior Frontal Gyrus | 44 | -52 | 10 | 14 | 4.67 |
|  |  | Right | Sub-Gyral | 6 | 28 | 0 | 54 | 4.59 |
|  |  | Right | Thalamus |  | 18 | -10 | 12 | 4.58 |
|  |  | Left | Insula | 13 | -38 | 10 | 8 | 4.57 |
|  |  | Right | Inferior Parietal Lobule | 40 | 36 | -44 | 44 | 4.50 |
|  |  | Left | Middle Frontal Gyrus | 6 | -24 | -2 | 60 | 4.45 |
|  |  | Right | Superior Parietal Lobule | 7 | 28 | -62 | 50 | 4.35 |
|  |  | Right | Superior Parietal Lobule | 7 | 34 | -50 | 46 | 4.33 |
|  |  | Right | Superior Parietal Lobule | 7 | 32 | -50 | 50 | 4.30 |
|  |  | Left | Thalamus |  | -12 | -4 | 12 | 4.30 |
|  |  | Right | Precuneus | 7 | 24 | -66 | 50 | 4.27 |
|  |  | Right | Superior Parietal Lobule | 7 | 32 | -62 | 46 | 4.26 |
|  |  | Left | Middle Frontal Gyrus | 6 | -30 | 2 | 54 | 4.25 |
|  |  | Left | Middle Frontal Gyrus | 6 | -36 | 2 | 52 | 4.24 |
|  |  | Left | Inferior Parietal Lobule | 40 | -44 | -36 | 50 | 4.20 |
|  |  | Left | Precentral Gyrus | 4 | -38 | -8 | 58 | 4.13 |
|  |  | Right | Middle Frontal Gyrus | 46 | 44 | 40 | 18 | 4.09 |
|  |  | Right | Middle Frontal Gyrus | 6 | 28 | -6 | 60 | 4.07 |
|  |  | Left | Precentral Gyrus | 4 | -38 | -12 | 56 | 4.04 |
|  |  | Right | Middle Frontal Gyrus | 46 | 50 | 38 | 10 | 4.01 |
|  |  | Right | Superior Frontal Gyrus | 9 | 38 | 42 | 26 | 4.00 |
|  |  | Right | Inferior Parietal Lobule | 40 | 52 | -40 | 50 | 3.88 |
|  |  | Right | Middle Frontal Gyrus | 9 | 48 | 24 | 26 | 3.81 |
|  |  | Right | Lentiform Nucleus |  | 14 | 4 | 0 | 3.80 |
|  |  | Left | Superior Temporal Gyrus | 13 | -50 | -20 | 6 | 3.73 |
|  |  | Left | Superior Frontal Gyrus | 10 | -32 | 56 | 16 | 3.71 |
|  |  | Left | Superior Temporal Gyrus | 41 | -56 | -18 | 6 | 3.66 |
|  |  | Left | Middle Frontal Gyrus | 9 | -46 | 20 | 36 | 3.63 |
|  |  | Right |  |  | 12 | -18 | -12 | 3.53 |
|  |  | Left | Inferior Parietal Lobule | 40 | -52 | -34 | 36 | 3.53 |
|  |  | Right | Superior Temporal Gyrus | 22 | 52 | -32 | 0 | 3.51 |
|  |  | Right | Transverse Temporal Gyrus | 41 | 50 | -18 | 10 | 3.48 |
|  |  | Left | Inferior Parietal Lobule | 40 | -46 | -50 | 50 | 3.42 |
|  |  | Right | Precuneus | 7 | 8 | -64 | 50 | 3.35 |
|  |  | Right | Insula | 13 | 38 | 8 | 8 | 3.33 |
|  |  | Left | Parahippocampal Gyrus |  | -22 | -6 | -16 | 3.29 |
|  |  | Left | Postcentral Gyrus | 3 | -40 | -24 | 56 | 3.29 |
|  |  | Left | Parahippocampal Gyrus | 30 | -16 | -32 | -6 | 3.21 |
|  |  | Left | Parahippocampal Gyrus | 30 | -14 | -32 | -10 | 3.14 |
|  |  | Left | Precuneus | 7 | 0 | -74 | 46 | 3.12 |
|  |  | Right | Insula | 13 | 42 | 0 | 6 | 3.10 |
|  |  | Left | Parahippocampal Gyrus | 28 | -22 | -20 | -18 | 3.08 |
|  |  | Right | Superior Temporal Gyrus | 22 | 56 | -16 | 6 | 3.03 |
|  |  | Left | Inferior Parietal Lobule | 40 | -58 | -22 | 34 | 3.00 |
|  |  | Left | Thalamus |  | -12 | -20 | 18 | 2.95 |
|  |  | Left | Postcentral Gyrus | 40 | -60 | -20 | 22 | 2.94 |
|  |  | Left | Postcentral Gyrus | 40 | -62 | -18 | 18 | 2.93 |
|  |  | Right | Superior Temporal Gyrus | 22 | 66 | -28 | 4 | 2.92 |
|  |  | Left | Insula | 13 | -40 | -6 | -2 | 2.92 |
|  |  | Left |  |  | -2 | -26 | -10 | 2.91 |
|  |  | Left | Parahippocampal Gyrus | 28 | -20 | -26 | -12 | 2.91 |
|  |  | Right | Superior Frontal Gyrus | 9 | 32 | 52 | 28 | 2.81 |
|  |  | Right |  |  | 4 | -26 | -14 | 2.81 |
|  |  | Left | Superior Temporal Gyrus | 22 | -46 | -18 | -8 | 2.78 |
|  |  | Right | Middle Frontal Gyrus | 9 | 48 | 38 | 28 | 2.72 |
|  |  | Left | Inferior Parietal Lobule | 40 | -60 | -30 | 24 | 2.64 |
|  |  | Right | Culmen |  | 12 | -34 | -8 | 2.63 |
|  |  | Right | Lentiform Nucleus |  | 28 | -14 | 8 | 2.58 |
|  |  | Right | Superior Temporal Gyrus | 41 | 62 | -14 | 4 | 2.55 |
|  |  | Right | Cuneus | 19 | 6 | -76 | 38 | 2.51 |
|  |  | Right | Postcentral Gyrus | 2 | 62 | -20 | 36 | 2.47 |
|  |  | Left |  |  | -10 | -18 | -8 | 2.46 |
|  |  | Right | Middle Frontal Gyrus | 10 | 36 | 54 | 10 | 2.42 |
|  |  | Right | Superior Frontal Gyrus | 9 | 22 | 48 | 24 | 2.41 |
|  |  | Right | Insula | 13 | 42 | -4 | 14 | 2.39 |
|  |  | Right | Parahippocampal Gyrus | 28 | 20 | -14 | -20 | 2.37 |
|  |  | Right | Parahippocampal Gyrus |  | 22 | -10 | -18 | 2.29 |
|  |  | Right | Middle Frontal Gyrus | 6 | 30 | 16 | 50 | 2.16 |

Abbreviations: BA, Brodmann area. Coordinates are Coordinates are MNI152 standard stereotaxic spaces.
