# Supplementary material for: The shared neural substrates of emotional mimicry and emotional contagion: an activation likelihood estimation meta-analysis and meta-analytic connectivity modeling analysis
Source: Soc Cogn Affect Neurosci. 2025 Sep 10;20(1):nsaf091. doi: 10.1093/scan/nsaf091 (PMC12542505; doi:10.1093/scan/nsaf091)
Supplement: nsaf091_Supplementary_Data [file nsaf091_supplementary_data.zip › scan-24-149-File013.docx]

Supplementary Materials

Supplementary Tables

Supplementary Table 1

Characters of studies included in the meta-analysis for emotional mimicry and emotional contagion.

| Reference | Sample size | Mean age | Number of coordinates | Coordinate space | Contrast |
| --- | --- | --- | --- | --- | --- |
| **Emotional mimicry** |  |  |  |  |  |
|  |  |  |  |  |  |
| Miyata et al., 2021 | 16 | 22.42 | 52 | MNI | Emotional mimicry vs Observing |
| Krautheim et al., 2020 | 178 | 24.04 | 38 | MNI | Emotional mimicry vs Observing |
| Numata et al., 2020 | 39 | 21.49 | 14 | MNI | Emotional imitation vs No imitation |
| Pohl et al., 2017 | 13 | —— | 9 | MNI | Emotional mimicry vs Observing |
| Christov‐Moore & Iacoboni, 2016 | 20 | 18~35 | 15 | MNI | Emotional mimicry vs Observing |
| Budell et al., 2015 | 23 | 18~33 | 9 | Talairach | Imitate vs Generate emotion |
| Braadbaart et al., 2014 | 20 | 26.3 | 4 | MNI | Emotional mimicry vs Mouth movement |
| Horan et al., 2014 | 23 | 18~60 | 6 | MNI | Emotional mimicry vs Executing |
|  |  |  | 6 |  | Emotional mimicry vs Observing |
| Lenzi et al., 2013 | 23 | 20~28 | 33 | MNI | Emotional mimicry vs Observing |
| Pohl et al., 2013 | 27 | 24.6 | 1 | MNI | Emotional mimicry vs Observing |
| Vrticka et al., 2013 | 20 | 33.5±4.5 | 3 | MNI | Emotional imitation vs Gender |
|  |  |  | 7 |  | Emotional mimicry vs Expressive suppression |
| Hennenlotter et al., 2009 | 19 | —— | 37 | Talairach | Emotional mimicry vs Rest |
| Van der Gaag et al., 2007 | 17 | 23.3 | 57 | MNI | Emotional mimicry vs Rest |
|  |  |  | 35 |  | Emotional imitation vs No imitation |
| Dapretto et al., 2006 | 10 | 12.38 | 36 | Talairach | Emotional mimicry vs Rest |
| Lee et al., 2006 | 18 | 26 | 19 | MNI | Emotional mimicry vs Observing |
|  |  |  | 32 |  | Mimicry: Emotional vs Ingestive |
| Carr et al., 2003 | 11 | 29 | 39 | Talairach | Emotional mimicry vs Observing |
| **Emotional contagion** |  |  |  |  |  |
|  |  |  |  |  |  |
| Hsu et al., 2022 | 14 | 22.82 ± 2.59 | 10 | MNI | Passive observing：Emotional vs Neutral |
| Tamm et al., 2020 | 69 | 20~30&65~75 | 107 | MNI | Passive observing：Emotional vs Neutral |
| Härtwig et al., 2020 | 26 | 34.69 | 1 | MNI | Emotional empathy vs Neutral |
| Krautheim et al., 2019 | 178 | 24 | 12 | MNI | Perception: Emotional face vs Neutral face |
| Rymarczyk et al.,2019 | 56 | 23.8 | 66 | MNI | Passive observing：Emotional vs Neutral |
| Oliver et al., 2018 | 36 | 21.5 | 14 | MNI | Emotional empathy vs Age estimation |
| Rymarczyk et al.,2018 | 47 | 23.7 | 61 | MNI | Passive observing：Emotional vs Neutral |
| Pohl et al., 2017 | 13 | —— | 9 | MNI | Imitation and observing：Emotional vs Neutral |
| Lenzi et al., 2016 | 30 | 31.5 | 14 | MNI | Passive observing：Emotional vs Neutral |
|  |  |  | 33 |  | Emotional empathy vs Rest |
| Mathur et al., 2016 | 15 | 25.3 | 21 | MNI | Passive observing：Emotional vs Neutral |
| Harada,2016 | 18 | 22.2 | 18 | Talairach | Happay target > Neutral non-target |
| Seara-Cardoso et al., 2016 | 30 | 26.9 | 11 | MNI | Emotional empathy vs Passive observing |
| Mazza et al., 2015 | 10 | 30 | 2 | Talairach | Implicit empathy: Emotional vs Neutral |
|  |  |  | 2 |  | Explicit empathy: Emotional vs Neutral |
| Singh et al., 2015 | 14 | 27.21 | 14 | MNI | Passive observing：Emotional vs Mosaic |
| Kanske et al., 2015 | 178 | 40.9 | 23 | MNI | Passive observing：Emotional vs Neutral |
|  | 25 | 32.6 | 16 |  |  |
| Lenzi et al., 2013 | 23 | 23.45 | 35 | MNI | Emotional empathy vs Rest |
| Ernst et al., 2013 | 18 | 27 | 5 | MNI | Passive observing：Emotional vs Mosaic |
| Fehr,2014 | 20 | 24.6 | 73 | Talairach | Passive observing：Emotional vs Neutral |
| Hadjikhani et al., 2014 | 31 | 22.5 | 41 | MNI | Passive observing：Emotional vs Mosaic |
| de Greck et al., 2012 | 32 | 23.1 | 25 | Talairach | Emotional empathy > Skin color evaluation |
| de Greck et al., 2012 | 20 | 37 | 29 | Talairach | Emotional empathy vs Unrecognizable content |
| Fan et al., 2011 | 20 | 40.18 | 23 | MNI | Passive observing：Emotional vs control |
| Schulte-Rüther et al., 2011 | 18 | 25.1 | 17 | MNI | Emotional empathy vs Evaluating picture’s width |
| Schnell et al., 2010 | 28 | 25.49 | 13 | MNI | Emotional empathy vs visuospatial judgements |
| Greimel et al., 2010 | 47 | 16.1 | 45 | MNI | Passive observing：Emotional vs Neutral |
| Nomi et al., 2008 | 14 | 28.6 | 6 | MNI | Emotional empathy vs Mosaic |
| Nummenmaa et al.,2008 | 20 | 26 | 8 | MNI | Passive observing：Emotional vs Neutral |
| Seitz et al.,2008 | 14 | 28.6 | 7 | MNI | Generate emotion vs Counting items |
|  |  |  | 18 |  | Passive observing： Emotional vs Mosaic |
| Schulte-Ruether et al., 2007 | 26 | 24.6 | 13 | MNI | Observing vs baseline |
| Dapretto et al., 2006 | 10 | 12.38 | 14 | Talairach | Observing vs rest |
| Lee et al., 2006 | 18 | 26 | 7 | MNI | Passive observing：Emotional vs Neutral |
