# Supplementary material for: The shared neural substrates of emotional mimicry and emotional contagion: an activation likelihood estimation meta-analysis and meta-analytic connectivity modeling analysis
Source: Soc Cogn Affect Neurosci. 2025 Sep 10;20(1):nsaf091. doi: 10.1093/scan/nsaf091 (PMC12542505; doi:10.1093/scan/nsaf091)
Supplement: nsaf091_Supplementary_Data [file nsaf091_supplementary_data.zip › scan-24-149-File017.docx]

Supplementary Materials

Supplementary Tables

Supplementary Table 5

MACM results: areas of functional coactivation associated with cluster 4(Right cerebrum: precentral gyrus).

| Cluster | Volume | Hemisphere | Region | BA(s) | Coordinate | | | ALE |
| --- | --- | --- | --- | --- | --- | --- | --- | --- |
|  | (mm^3^) |  |  |  | x | y | z | （×10^-2^） |
| 1 | 53896 | Left | Insula | 13 | -34 | 26 | -2 | 3.63 |
|  |  | Left | Parahippocampal Gyrus |  | -18 | -6 | -18 | 3.16 |
|  |  | Left | Claustrum |  | -34 | 18 | 0 | 3.10 |
|  |  | Left | Insula | 13 | -30 | 22 | 8 | 2.82 |
|  |  | Left | Precentral Gyrus | 6 | -46 | 2 | 40 | 2.76 |
|  |  | Right | Thalamus |  | 6 | -14 | 6 | 2.71 |
|  |  | Left | Precentral Gyrus | 6 | -34 | -2 | 52 | 2.40 |
|  |  | Left | Precentral Gyrus | 6 | -46 | -4 | 50 | 2.40 |
|  |  | Left | Middle Frontal Gyrus | 6 | -36 | 0 | 48 | 2.39 |
|  |  | Left | Thalamus |  | -10 | -12 | 4 | 2.30 |
|  |  | Left | Middle Frontal Gyrus | 46 | -46 | 34 | 16 | 2.13 |
|  |  | Left | Precentral Gyrus | 44 | -50 | 6 | 4 | 2.02 |
|  |  | Left | Middle Frontal Gyrus | 6 | -26 | -2 | 58 | 2.02 |
|  |  | Left | Inferior Frontal Gyrus | 9 | -50 | 12 | 22 | 2.01 |
|  |  | Right | Lentiform Nucleus |  | 22 | 4 | 0 | 1.99 |
|  |  | Right | Lentiform Nucleus |  | 16 | 4 | -4 | 1.98 |
|  |  | Right | Thalamus |  | 14 | -8 | 8 | 1.98 |
|  |  | Left | Precentral Gyrus | 44 | -60 | 12 | 10 | 1.97 |
|  |  | Right | Thalamus |  | 8 | -4 | -2 | 1.89 |
|  |  | Left | Middle Frontal Gyrus | 46 | -48 | 32 | 22 | 1.81 |
|  |  | Left | Lentiform Nucleus |  | -26 | 2 | 4 | 1.79 |
|  |  | Left | Middle Frontal Gyrus | 10 | -32 | 46 | 20 | 1.75 |
|  |  | Left | Lentiform Nucleus |  | -24 | 10 | 4 | 1.73 |
|  |  | Left | Middle Frontal Gyrus | 9 | -38 | 38 | 22 | 1.70 |
|  |  | Left | Superior Frontal Gyrus | 9 | -30 | 48 | 24 | 1.66 |
|  |  | Left | Thalamus |  | -18 | -6 | 10 | 1.64 |
|  |  | Left | Lentiform Nucleus |  | -20 | 4 | -4 | 1.58 |
|  |  | Left | Precentral Gyrus | 6 | -60 | 6 | 20 | 1.57 |
|  |  | Right |  |  | 12 | -18 | -6 | 1.44 |
|  |  | Left | Parahippocampal Gyrus | 28 | -22 | -18 | -18 | 1.41 |
|  |  | Left | Insula | 13 | -44 | -4 | 12 | 1.31 |
|  |  | Left | Precentral Gyrus | 6 | -56 | -4 | 24 | 1.28 |
|  |  | Right | Caudate |  | 18 | 2 | 14 | 1.21 |
|  |  | Left |  |  | -8 | -18 | -6 | 1.08 |
|  |  | Left | Caudate |  | -14 | 4 | 12 | 1.02 |
| 2 | 19032 | Right | Precentral Gyrus | 6 | 50 | 2 | 44 | 9.44 |
|  |  | Right | Inferior Frontal Gyrus | 9 | 46 | 8 | 26 | 2.51 |
|  |  | Right | Precentral Gyrus | 6 | 38 | -6 | 58 | 2.34 |
|  |  | Right | Middle Frontal Gyrus | 6 | 24 | -4 | 62 | 2.20 |
|  |  | Right | Inferior Frontal Gyrus | 9 | 62 | 10 | 26 | 1.96 |
|  |  | Right | Middle Frontal Gyrus | 6 | 32 | -2 | 48 | 1.91 |
|  |  | Right | Inferior Frontal Gyrus | 44 | 56 | 12 | 16 | 1.75 |
|  |  | Right | Middle Frontal Gyrus | 46 | 60 | 28 | 18 | 1.71 |
|  |  | Right | Superior Temporal Gyrus | 22 | 62 | -8 | -6 | 1.53 |
|  |  | Right | Superior Temporal Gyrus | 22 | 52 | -22 | -2 | 1.52 |
|  |  | Right | Superior Temporal Gyrus |  | 58 | -20 | -2 | 1.45 |
|  |  | Right | Superior Temporal Gyrus | 41 | 60 | -22 | 2 | 1.40 |
|  |  | Right | Superior Temporal Gyrus | 22 | 58 | 6 | -2 | 1.34 |
|  |  | Right | Superior Temporal Gyrus | 22 | 56 | 6 | 2 | 1.31 |
|  |  | Right | Superior Temporal Gyrus | 22 | 60 | -2 | 2 | 1.22 |
|  |  | Right | Middle Frontal Gyrus | 46 | 48 | 24 | 16 | 1.16 |
| 3 | 17864 | Left | Superior Frontal Gyrus | 6 | -4 | 12 | 58 | 3.81 |
|  |  | Left | Medial Frontal Gyrus | 6 | 2 | 0 | 62 | 3.21 |
|  |  | Left | Medial Frontal Gyrus | 6 | -6 | -6 | 66 | 2.89 |
|  |  | Right | Cingulate Gyrus | 32 | 8 | 16 | 40 | 2.49 |
|  |  | Right | Medial Frontal Gyrus | 32 | 10 | 16 | 46 | 2.44 |
|  |  | Left | Medial Frontal Gyrus | 6 | 0 | 2 | 50 | 2.37 |
|  |  | Left | Cingulate Gyrus | 24 | -4 | 4 | 38 | 1.89 |
|  |  | Left | Medial Frontal Gyrus | 6 | -2 | 24 | 46 | 1.35 |
|  |  | Right | Cingulate Gyrus | 32 | 8 | 24 | 28 | 1.14 |
|  |  | Right | Cingulate Gyrus | 32 | 6 | 28 | 36 | 1.05 |

Abbreviations: BA, Brodmann area. Coordinates are Coordinates are MNI152 standard stereotaxic spaces.
