# Supplementary material for: The shared neural substrates of emotional mimicry and emotional contagion: an activation likelihood estimation meta-analysis and meta-analytic connectivity modeling analysis
Source: Soc Cogn Affect Neurosci. 2025 Sep 10;20(1):nsaf091. doi: 10.1093/scan/nsaf091 (PMC12542505; doi:10.1093/scan/nsaf091)
Supplement: nsaf091_Supplementary_Data [file nsaf091_supplementary_data.zip › scan-24-149-File014.docx]

Supplementary Materials

Supplementary Tables

Supplementary Table 2

MACM results: areas of functional coactivation associated with cluster1 ((Left cerebrum: cingulate gyrus).

| Cluster | Volume | Hemisphere | Region | BA(s) | Coordinate | | | ALE |
| --- | --- | --- | --- | --- | --- | --- | --- | --- |
|  | (mm^3^) |  |  |  | x | y | z | （×10^-2^） |
| 1 | 221424 | Left | Insula | 13 | -34 | 24 | 0 | 12.72 |
|  |  | Right | Insula | 13 | 34 | 22 | 4 | 12.17 |
|  |  | Right | Insula |  | 38 | 22 | -4 | 10.62 |
|  |  | Right | Thalamus |  | 12 | -16 | 6 | 9.74 |
|  |  | Left | Insula | 13 | -38 | 18 | -8 | 9.70 |
|  |  | Left | Thalamus |  | -10 | -16 | 4 | 9.63 |
|  |  | Left | Precentral Gyrus | 6 | -50 | 2 | 38 | 9.26 |
|  |  | Left | Inferior Frontal Gyrus | 9 | -44 | 12 | 24 | 9.02 |
|  |  | Left | Inferior Parietal Lobule | 40 | -58 | -30 | 24 | 8.49 |
|  |  | Left | Lentiform Nucleus |  | -22 | 8 | 4 | 8.40 |
|  |  | Right | Lentiform Nucleus |  | 24 | 2 | 4 | 7.94 |
|  |  | Left | Inferior Frontal Gyrus | 44 | -54 | 8 | 20 | 7.59 |
|  |  | Left | Inferior Frontal Gyrus | 9 | -50 | 2 | 26 | 7.49 |
|  |  | Right | Precentral Gyrus | 6 | 52 | 0 | 40 | 7.46 |
|  |  | Left | Thalamus | Nucleus | -14 | -4 | 10 | 7.44 |
|  |  | Right | Inferior Frontal Gyrus | 9 | 48 | 8 | 28 | 7.27 |
|  |  | Left | Precentral Gyrus | 6 | -54 | -4 | 26 | 7.11 |
|  |  | Right | Superior Parietal Lobule | 7 | 30 | -62 | 46 | 6.95 |
|  |  | Left | Precentral Gyrus | 6 | -28 | -4 | 52 | 6.88 |
|  |  | Left | Precentral Gyrus | 6 | -36 | -4 | 56 | 6.79 |
|  |  | Right | Precentral Gyrus | 6 | 54 | 6 | 34 | 6.73 |
|  |  | Right | Thalamus |  | 8 | -6 | 6 | 6.45 |
|  |  | Right | Precentral Gyrus | 6 | 36 | -2 | 54 | 6.33 |
|  |  | Left | Superior Parietal Lobule | 7 | -28 | -54 | 52 | 6.21 |
|  |  | Left | Middle Frontal Gyrus | 46 | -46 | 30 | 18 | 6.20 |
|  |  | Left | Inferior Parietal Lobule | 40 | -34 | -48 | 44 | 6.07 |
|  |  | Right | Middle Frontal Gyrus | 9 | 46 | 18 | 24 | 6.04 |
|  |  | Left | Claustrum |  | -32 | -12 | 12 | 6.01 |
|  |  | Left | Inferior Parietal Lobule | 40 | -36 | -44 | 44 | 5.99 |
|  |  | Left | Superior Parietal Lobule | 7 | -26 | -60 | 50 | 5.98 |
|  |  | Left | Middle Frontal Gyrus | 9 | -38 | 38 | 30 | 5.85 |
|  |  | Right | Superior Frontal Gyrus | 9 | 38 | 44 | 26 | 5.71 |
|  |  | Right | Inferior Parietal Lobule | 40 | 42 | -44 | 46 | 5.62 |
|  |  | Left | Claustrum |  | -34 | 0 | 10 | 5.54 |
|  |  | Left | Inferior Parietal Lobule | 40 | -48 | -32 | 42 | 5.46 |
|  |  | Left | Middle Temporal Gyrus | 22 | -50 | -40 | 12 | 5.33 |
|  |  | Left | Inferior Parietal Lobule | 40 | -48 | -36 | 42 | 5.27 |
|  |  | Right | Superior Frontal Gyrus | 9 | 44 | 40 | 24 | 5.25 |
|  |  | Left | Precentral Gyrus | 6 | -50 | -2 | 4 | 5.18 |
|  |  | Right | Inferior Parietal Lobule | 40 | 64 | -32 | 22 | 5.12 |
|  |  | Right | Superior Temporal Gyrus | 22 | 56 | -8 | -4 | 5.07 |
|  |  | Left | Transverse Temporal Gyrus | 42 | -60 | -14 | 14 | 5.02 |
|  |  | Right | Superior Temporal Gyrus | 41 | 56 | -26 | 4 | 4.98 |
|  |  | Right | Inferior Parietal Lobule | 40 | 60 | -28 | 24 | 4.92 |
|  |  | Left | Precuneus | 7 | -20 | -72 | 48 | 4.90 |
|  |  | Right | Middle Frontal Gyrus | 9 | 44 | 28 | 28 | 4.85 |
|  |  | Left | Precuneus | 7 | -18 | -76 | 50 | 4.82 |
|  |  | Right | Precentral Gyrus | 44 | 58 | 14 | 2 | 4.81 |
|  |  | Left | Superior Temporal Gyrus | 41 | -58 | -26 | 8 | 4.79 |
|  |  | Left |  | Body | -22 | -28 | -6 | 4.77 |
|  |  | Left | Superior Temporal Gyrus | 22 | -48 | -36 | 6 | 4.72 |
|  |  | Right | Insula | 40 | 56 | -20 | 12 | 4.72 |
|  |  | Left | Precuneus | 7 | -18 | -64 | 58 | 4.70 |
|  |  | Right | Superior Temporal Gyrus | 39 | 52 | -50 | 12 | 4.62 |
|  |  | Right | Postcentral Gyrus | 43 | 60 | -16 | 14 | 4.61 |
|  |  | Right | Inferior Parietal Lobule | 40 | 62 | -38 | 28 | 4.57 |
|  |  | Left | Precuneus | 19 | -22 | -70 | 42 | 4.46 |
|  |  | Left | Precentral Gyrus | 4 | -36 | -16 | 58 | 4.46 |
|  |  | Right | Postcentral Gyrus | 2 | 60 | -24 | 40 | 4.46 |
|  |  | Right | Superior Parietal Lobule | 7 | 22 | -62 | 62 | 4.44 |
|  |  | Right | Precuneus | 31 | 30 | -74 | 26 | 4.43 |
|  |  | Right | Precuneus | 31 | 30 | -72 | 30 | 4.36 |
|  |  | Right | Middle Temporal Gyrus | 22 | 58 | -52 | 4 | 4.27 |
|  |  | Right | Precentral Gyrus | 6 | 60 | 4 | 4 | 4.27 |
|  |  | Right | Supramarginal Gyrus | 40 | 60 | -44 | 34 | 4.26 |
|  |  | Left | Inferior Parietal Lobule | 40 | -42 | -52 | 56 | 4.15 |
|  |  | Right | Inferior Parietal Lobule | 40 | 52 | -32 | 44 | 4.07 |
|  |  | Right | Precuneus | 7 | 14 | -74 | 50 | 4.02 |
|  |  | Left | Insula | 13 | -44 | -18 | 18 | 3.83 |
|  |  | Left | Transverse Temporal Gyrus | 41 | -42 | -32 | 14 | 3.68 |
|  |  | Right | Lentiform Nucleus |  | 22 | 0 | -14 | 3.65 |
|  |  | Right | Middle Frontal Gyrus | 8 | 30 | 26 | 40 | 3.56 |
|  |  | Right | Precentral Gyrus | 4 | 44 | -14 | 44 | 3.04 |

Abbreviations: BA, Brodmann area. Coordinates are Coordinates are MNI152 standard stereotaxic spaces.
