# Supplementary material for: The shared neural substrates of emotional mimicry and emotional contagion: an activation likelihood estimation meta-analysis and meta-analytic connectivity modeling analysis
Source: Soc Cogn Affect Neurosci. 2025 Sep 10;20(1):nsaf091. doi: 10.1093/scan/nsaf091 (PMC12542505; doi:10.1093/scan/nsaf091)
Supplement: nsaf091_Supplementary_Data [file nsaf091_supplementary_data.zip › scan-24-149-File016.docx]

Supplementary Materials

Supplementary Tables

Supplementary Table 4

MACM results: areas of functional coactivation associated with cluster 3(Right cerebrum: inferior parietal lobule).

| Cluster | Volume | Hemisphere | Region | BA(s) | Coordinate | | | ALE |
| --- | --- | --- | --- | --- | --- | --- | --- | --- |
|  | (mm^3^) |  |  |  | x | y | z | （×10^-2^） |
| 1 | 111584 | Right | Inferior Parietal Lobule | 40 | 58 | -28 | 26 | 12.36 |
|  |  | Right | Thalamus |  | 10 | -14 | 6 | 4.94 |
|  |  | Left | Postcentral Gyrus | 40 | -60 | -30 | 22 | 4.40 |
|  |  | Left | Inferior Parietal Lobule | 40 | -56 | -32 | 32 | 4.04 |
|  |  | Right | Insula | 13 | 46 | 6 | 4 | 3.32 |
|  |  | Left | Insula | 13 | -38 | 4 | 6 | 3.27 |
|  |  | Right | Insula | 13 | 40 | -2 | 14 | 3.20 |
|  |  | Left | Thalamus |  | -12 | -18 | 8 | 3.12 |
|  |  | Left | Superior Temporal Gyrus | 22 | -56 | 6 | 4 | 2.99 |
|  |  | Right | Inferior Frontal Gyrus | 44 | 56 | 10 | 10 | 2.98 |
|  |  | Right | Middle Frontal Gyrus | 6 | 34 | -2 | 62 | 2.95 |
|  |  | Left | Postcentral Gyrus | 40 | -62 | -20 | 22 | 2.83 |
|  |  | Left | Thalamus |  | -20 | -14 | 6 | 2.79 |
|  |  | Left | Thalamus |  | -14 | -18 | 0 | 2.75 |
|  |  | Left | Insula | 13 | -44 | 8 | -6 | 2.74 |
|  |  | Right | Precentral Gyrus | 6 | 46 | -2 | 46 | 2.73 |
|  |  | Right | Lentiform Nucleus |  | 26 | 4 | 6 | 2.56 |
|  |  | Right | Precentral Gyrus | 6 | 54 | -4 | 36 | 2.46 |
|  |  | Left | Lentiform Nucleus |  | -12 | 6 | 0 | 2.45 |
|  |  | Left | Insula | 13 | -42 | -4 | 8 | 2.44 |
|  |  | Left |  |  | -6 | -22 | -10 | 2.42 |
|  |  | Left | Precentral Gyrus | 43 | -56 | -8 | 10 | 2.40 |
|  |  | Right | Caudate |  | 12 | 6 | 2 | 2.37 |
|  |  | Left | Lentiform Nucleus |  | -24 | 0 | -6 | 2.36 |
|  |  | Left | Thalamus |  | -20 | -28 | -2 | 2.32 |
|  |  | Right | Precentral Gyrus | 6 | 54 | 6 | 30 | 2.25 |
|  |  | Right | Claustrum |  | 36 | 12 | 6 | 2.24 |
|  |  | Right | Lentiform Nucleus |  | 26 | -2 | 0 | 2.24 |
|  |  | Right | Precentral Gyrus | 6 | 56 | -2 | 30 | 2.22 |
|  |  | Left | Precentral Gyrus | 6 | -50 | -2 | 42 | 2.21 |
|  |  | Right |  |  | 6 | -26 | -12 | 2.16 |
|  |  | Right | Lentiform Nucleus |  | 26 | -2 | -10 | 2.14 |
|  |  | Right | Inferior Frontal Gyrus | 47 | 50 | 20 | -16 | 2.14 |
|  |  | Left | Lentiform Nucleus |  | -24 | -6 | -12 | 2.11 |
|  |  | Left | Precentral Gyrus | 6 | -54 | -8 | 32 | 2.08 |
|  |  | Left | Lentiform Nucleus |  | -24 | -6 | -8 | 2.07 |
|  |  | Left | Insula | 13 | -40 | -18 | 12 | 2.06 |
|  |  | Right | Superior Temporal Gyrus | 41 | 60 | -18 | 4 | 2.02 |
|  |  | Left |  |  | 0 | -28 | -16 | 2.02 |
|  |  | Right | Claustrum |  | 32 | 22 | 4 | 2.01 |
|  |  | Left | Claustrum |  | -30 | -14 | 14 | 2.00 |
|  |  | Left | Lentiform Nucleus |  | -24 | 6 | 6 | 1.94 |
|  |  | Right | Insula | 13 | 48 | -16 | 10 | 1.91 |
|  |  | Left | Parahippocampal Gyrus | 34 | -22 | -12 | -20 | 1.91 |
|  |  | Left | Postcentral Gyrus | 3 | -60 | -8 | 22 | 1.84 |
|  |  | Right | Superior Temporal Gyrus | 22 | 58 | 8 | -4 | 1.78 |
|  |  | Left | Inferior Frontal Gyrus | 47 | -48 | 16 | -10 | 1.77 |
|  |  | Right | Lentiform Nucleus |  | 26 | 6 | -4 | 1.74 |
|  |  | Right | Inferior Parietal Lobule | 40 | 54 | -26 | 50 | 1.70 |
|  |  | Right | Precentral Gyrus | 4 | 44 | -12 | 42 | 1.70 |
|  |  | Right | Middle Frontal Gyrus | 6 | 52 | 12 | 42 | 1.68 |
|  |  | Left | Middle Temporal Gyrus | 22 | -54 | -44 | 6 | 1.66 |
|  |  | Left | Caudate |  | -10 | 2 | 10 | 1.62 |
|  |  | Left | Postcentral Gyrus | 3 | -48 | -16 | 40 | 1.58 |
|  |  | Left | Thalamus |  | -4 | -6 | -2 | 1.57 |
|  |  | Right | Precentral Gyrus | 6 | 44 | -10 | 36 | 1.56 |
|  |  | Left | Inferior Parietal Lobule | 40 | -60 | -44 | 24 | 1.53 |
|  |  | Right | Precentral Gyrus | 6 | 42 | -6 | 32 | 1.51 |
|  |  | Right | Parahippocampal Gyrus | 28 | 20 | -14 | -20 | 1.48 |
|  |  | Right | Inferior Frontal Gyrus | 47 | 42 | 26 | -18 | 1.42 |
|  |  | Left | Precentral Gyrus | 6 | -40 | -2 | 32 | 1.41 |
|  |  | Left |  |  | 2 | -16 | -14 | 1.31 |
|  |  | Left | Superior Temporal Gyrus | 39 | -52 | -56 | 8 | 1.26 |
| 2 | 19152 | Left | Medial Frontal Gyrus | 6 | 0 | -4 | 56 | 4.32 |
|  |  | Left | Medial Frontal Gyrus | 6 | 2 | 0 | 54 | 4.26 |
|  |  | Left | Cingulate Gyrus | 24 | 0 | 8 | 42 | 4.04 |
|  |  | Left | Cingulate Gyrus | 32 | 2 | 20 | 36 | 2.45 |
|  |  | Left | Cingulate Gyrus | 32 | -4 | 24 | 32 | 2.20 |
|  |  | Left | Medial Frontal Gyrus | 6 | -10 | -2 | 70 | 1.92 |
|  |  | Right | Medial Frontal Gyrus | 6 | 8 | -8 | 74 | 1.53 |
|  |  | Right | Cingulate Gyrus | 32 | 4 | 34 | 26 | 1.44 |

Abbreviations: BA, Brodmann area. Coordinates are Coordinates are MNI152 standard stereotaxic spaces.
